# Cancer of unknown primary genomic profiling from cell-free DNA provides insights into CUP biology and vulnerabilities

**DOI:** 10.3389/fmed.2026.1777582

**Published:** 2026-06-03

**Authors:** Roberta Roncarati, Beatrice Fontana, Ilaria Pace, Giorgio Durante, Nicole Conci, Alessia Soru, Giulia Gallerani, Mattia Riefolo, Irene Salamon, Noemi Laprovitera, Elisabetta Broseghini, Maria Naddeo, Andrea Espis, Gabriele Sales, Stefano Diciotti, Antonia D’Errico, Karim Rihawi, Andrea Ardizzoni, Manuela Ferracin

**Affiliations:** 1 Department of Medical and Surgical Sciences (DIMEC), University of Bologna, Bologna, Italy; 2 CNR Institute of Molecular Genetics "Luigi Luca Cavalli-Sforza", Unit of Bologna, Bologna, Italy; 3 IRCCS Azienda Ospedaliero-Universitaria di Bologna, Bologna, Italy; 4 Dipartimento di Ingegneria dell'Energia Elettrica e dell'Informazione "Guglielmo Marconi" (DEI), University of Bologna, Bologna, Italy; 5 Department of Biology, University of Padova, Padua, Italy

**Keywords:** cancer genetics, cancer of unknown primary, CHIP, liquid biopsy, precision oncology, target therapy

## Abstract

**Introduction:**

Cancer of unknown primary (CUP) is a metastatic malignancy with no identifiable site of origin, accounting for 2–5% of cancer diagnoses. Its marked heterogeneity and the limited availability of tumor tissue pose major challenges to genomic profiling.

**Methods:**

In this retrospective observational study, we applied a 92-gene CUP-specific targeted sequencing panel to liquid biopsy samples from 39 CUP patients, analyzing circulating cell-free DNA (ccfDNA) together with paired germline DNA (gDNA), when available. Somatic, germline, and CHIP-related variants were classified using predefined variant allele frequency (VAF) thresholds and paired ccfDNA/gDNA comparison.

**Results:**

We identified somatic mutations in 78 of 92 genes and 44 clinically relevant variants (Tier I–III), most frequently affecting *NF1, KRAS, ARID1A,* and *PIK3CA*. Mutated genes were primarily involved in cell-cycle regulation, receptor tyrosine kinase signaling, and NOTCH pathways. Recurrent genetic alterations were detected in 15 genes, including canonical hotspot mutations in *TP53, KRAS, and PIK3CA*, 10 of which were shared by three or more patients. Actionable mutations, as defined by current clinical annotation guidelines, were identified in 13 genes, supporting the potential role of molecularly guided therapies in CUP. Likely CHIP-associated variants, defined as mutations in CHIP-associated genes with VAF ≥ 2% in PBMC-derived genomic DNA, were detected in 17 patients. In addition, pathogenic germline variants in *MITF, NTRK1, and BAP1* were identified in three patients; notably, the *NTRK1* germline variant was accompanied by an independent somatic mutation in the same gene.

**Discussion:**

Overall, these findings support liquid biopsy as a valuable approach for molecular profiling of CUP and highlight the critical importance of paired ccfDNA/gDNA analysis, including CHIP assessment, to accurately distinguish somatic, germline, and hematopoiesis-related variants.

## Background

Cancer of unknown primary (CUP) is a heterogeneous group of tumors that present as metastatic disease at diagnosis, for which the primary site cannot be identified despite extensive diagnostic procedures (1). CUP accounts for approximately 2–3% of newly diagnosed cancers worldwide and is associated with diverse clinical manifestations. Most patients present with metastases involving two or more organs at diagnosis (2, 3). CUP is characterized by an early propensity for metastasis, and the pattern of metastatic spread at presentation may provide clues regarding the possible site of origin. The prognosis of CUP patients is generally unfavorable, with approximately 30% surviving one year after diagnosis. Several risk factors have been associated with CUP development, including smoking, type 2 diabetes, autoimmune disorders, obesity, familial predisposition, and Black ethnicity (1, 4).

The diagnostic workup of CUP relies on a multidisciplinary approach that includes detailed medical history, physical examination, and extensive radiological evaluation, such as computed tomography (CT), positron emission tomography–CT (PET-CT), magnetic resonance imaging (MRI), and endoscopic examinations. Gynecological, dermatological, and ophthalmological assessments are also recommended when clinically indicated. Tumor biopsy is required for histopathological evaluation, immunohistochemistry (IHC), and, more recently, molecular and genomic testing (5). In addition, serum tumor markers (e.g., PSA, AFP, *β*-hCG, CA-125) may be included in the diagnostic workup (6).

According to international guidelines, CUPs are classified into favorable-prognosis subgroups (15–20% of cases), which are generally associated with longer survival (12–36 months), and poor-risk cases (80–85%), characterized by aggressive behavior and resistance to most therapies. Favorable prognostic features include specific clinicopathological entities, such as papillary carcinoma of the peritoneal cavity; characteristic metastatic patterns (e.g., axillary lymph node involvement from adenocarcinoma in women or inguinal lymph node involvement from squamous cell carcinoma); poorly differentiated neuroendocrine tumors; osteoblastic metastases with elevated PSA levels; and potentially resectable solitary lesions. Conversely, adverse prognostic factors include adenocarcinoma or undifferentiated carcinoma histology, male sex, Eastern Cooperative Oncology Group (ECOG) performance status >1, age >65 years, comorbidities, ascites, and multiple or critical metastatic sites (e.g., peritoneal, pleural, or brain involvement) (NCCN Guidelines Version 1.2026 (6)). Specifically, a prognostic model validated in a large multicenter cohort (*N* = 926) identified five independent predictors of worse outcomes: male sex, higher ECOG performance status, adenocarcinoma histology, number of metastatic sites, and elevated neutrophil-to-lymphocyte ratio, with ECOG performance status and neutrophil-to-lymphocyte ratio emerging as the strongest predictors (7).

Current international guidelines recommend empirical treatment strategies for CUP, most commonly platinum-based doublet chemotherapy as first-line therapy, tailored according to histological subtype (8–10). However, this approach highlights a substantial unmet clinical need for more effective and individualized treatment strategies.

In the clinical management of CUP patients, comprehensive genomic profiling is increasingly being evaluated in clinical trials to support molecularly guided therapy (1, 11). This approach could be further enhanced by integrating site-of-origin prediction methods based on gene expression profiling (12, 13), microRNA signatures (14, 15), or DNA methylation patterns (16, 17). Recent studies (18–20) have applied whole-genome and transcriptome sequencing combined with machine learning–based classifiers (such as CUPPA and CUPLR) to predict tissue of origin in CUP, achieving diagnostic accuracies ranging from ~58 to 78%, while also identifying actionable genomic alterations in up to 70–80% of patients. However, the limited availability of tumor tissue and the pronounced intratumoral heterogeneity of CUP pose significant challenges to tissue-based molecular testing. As a result, targeted molecular analyses of circulating cell-free DNA (ccfDNA) obtained from liquid biopsy samples have gained attention as a minimally invasive alternative for CUP molecular characterization (21).

The recently completed phase II, randomized, international CUPISCO trial evaluated the clinical benefit of next-generation sequencing (NGS)–guided targeted therapies in patients with histologically confirmed CUP (11). This study directly compared empirical platinum-based chemotherapy with molecularly matched therapies based on comprehensive genomic profiling. In addition, patients with microsatellite instability–high (MSI-H), mismatch repair–deficient (dMMR), or high tumor mutational burden (TMB-high) CUP tumors were considered for immune checkpoint inhibitor therapy. Based on the CUPISCO results, molecularly guided treatments may represent a promising second-line therapeutic option for patients with unfavorable CUP.

Blood-based liquid biopsy, particularly the analysis of circulating tumor DNA (ctDNA), has become an important tool in the management of advanced cancers, enabling the detection of tumor-derived genetic alterations for diagnostic, prognostic, and therapeutic purposes. However, accurate identification of tumor-derived ctDNA within the broader ccfDNA pool remains technically challenging. This challenge arises from the need to distinguish true tumor-specific alterations from germline variants and from somatic mutations associated with clonal hematopoiesis of indeterminate potential (CHIP). CHIP is an age-related clonal expansion of hematopoietic cells harboring somatic mutations, often affecting genes that are also common cancer drivers (22). Notably, the prevalence of CHIP increases with age and is associated with a 30–40% increase in all-cause mortality (23).

Given that the biological mechanisms underlying CUP initiation and progression remain incompletely understood, this study aimed to evaluate the utility of a liquid biopsy–based approach for the genetic characterization of CUP, with the goal of improving and supporting clinical management. By performing targeted sequencing of 92 cancer-related genes in ccfDNA from a cohort of CUP patients, we identified somatic genetic alterations not present in the general population, highlighting the marked genetic heterogeneity of CUP. Our analysis revealed the co-occurrence of multiple genetic alterations within individual patients, as well as recurrent alterations shared across different patients, including variants also detected at the germline level. Importantly, actionable molecular alterations with potential therapeutic relevance were identified. Furthermore, paired analysis of ccfDNA and matched genomic DNA enabled the identification of pathogenic germline variants and the detection of CHIP-related mutations, raising new questions regarding their potential role in CUP pathogenesis.

## Materials and methods

### Patients

Thirty-nine patients diagnosed with cancer of unknown primary (CUP) were recruited at the Oncology Unit of Bologna University Hospital, Italy, between 2017 and 2024. Patient characteristics are summarized in Table 1. The study was approved by the Comitato Etico Indipendente dell’Azienda Ospedaliero-Universitaria di Bologna, Policlinico S. Orsola-Malpighi (protocol number EM435-2022_130/2016/U/Tess/AOUBo). All patients provided written informed consent to participate in the study, and all procedures were conducted in accordance with relevant guidelines and regulations.

**Table 1:** Patient characteristics.

Patient	Cancer family history	Age at diagnosis	Tissue biopsy	Sex	Smoking history	Alcohol history consumption	Viral Infections	Histology	No of metastatic sites	Metastatic sites at diagnosis	ECOG performance status	Risk profile (U = unfavorable, F = favorable)	Chemotherapy	Immunotherapy	Best response
*N* = 39		Average = 65.5 (range 32–87)		M = 15; F = 24	Yes = 14; No = 18; NA = 7	Yes = 12; No = 20; NA = 7	HBV + = 4 HCV+ = 1 HPV+ = 3	Ca = 23; AD = 11; Others = 5	Average 2.3			*U* = 29, *F* = 6, NE = 4			SD = 6, PR = 9, PD = 14, NA = 10
CUP#009	Negative	40	Peritoneum and pleura	Female	NA	NA	*HBV - HCV - HPV -*	Carcinoma	3	Peritoneal carcinomatosis, lymph nodes, pleural effusion	0	U	Cisplatin + Gemcitabine	NA	PD
CUP#033	Unknown	76	Lymph node	Female	Yes	No	*HBV - HCV - HPV NA*	Carcinoma	3	Lymph nodes, lung, bone	1	U	Epirubicin + Cyclophosphamide	NA	SD
CUP#051	First-degree releative	78	Bone	Female	No	Yes	*HBV - HCV - HPV NA*	Carcinoma	3	Bone, liver, lymph nodes	1	U	Pazopanib	NA	PD
CUP#052	Unknown	82	Lung	Male	No	No	*HBV - HCV - HPV NA*	Melanoma	3	Bone, lung, brain	1	U	Zometa	Yes	PD
CUP#054	Unknown	59	Lung	Female	No	No	*HBV - HCV - HPV NA*	Carcinoma	5	Lung, lymph nodes, liver, breast, peritoneal carcinomatosis	0	U	Cisplatin + Gemcitabine	NA	SD
CUP#055	Third-degree relative: larynx cancer	50	Lymph node	Female	Yes	No	*HBV - HCV - HPV NA*	Adenocarcinoma	1	Lymph nodes	1	F	Folfox	NA	SD
CUP#062	First-degree releative: lung cancer	87	Prostate	Male	Yes	Yes	*HBV - HCV - HPV NA*	Carcinoma	3	Pelvis,seminal vesicles, peritoneal carcinomatosis	2	U	Folfox	NA	SD
CUP#063	Second-degree relative: HCC and cerebral cancer	68	Pleura	Male	Yes	Yes	*HBV - HCV - HPV NA*	Carcinoma	1	Pleura	1	U	Carboplatin + Gemcitabina	NA	PD
CUP#064	First-degree relative: uterine cancer	58	Liver	Male	Yes	Yes	*HBV - HCV - HPV NA*	Carcinoma	4	Lymph nodes, peritoneum, liver, bone	2	U	Cisplatin + 5-FU + Cetuximab	NA	PD
CUP#090	Negative	63	Lymph node	Female	No	No	*HBV + HCV - HPV NA*	Carcinoma	3	Lymph nodes, peritoneal carcinomatosis	1	U	Folfox	NA	PR
CUP#095	First-degree relative: lung cancer; other second-degree relatives:colon cancer	72	Soft tissue	Female	Yes	Yes	*HBV - HCV - HPV NA*	Carcinoma	2	Bone, soft tissue	2	U	Carboplatin + Taxolo	NA	PD
CUP#096	Third-degree relative: breast cancer	70	Ascitis	Female	No	Yes	*HBV - HCV - HPV NA*	Carcinoma	3	Peritoneal carcinomatosis, lymph nodes	3	U	NA	NA	NA
CUP#124	First-degree relative: lung cancer	70	Liver	Female	No	No	*HBV - HCV - HPV NA*	Adenocarcinoma	1	Liver	1	U	Gemcitabine + Oxaliplatin	NA	PR
CUP#125	Unknown	56	NA	Male	NA	NA	*HBV - HCV - HPV NA*	NA	NA	NA	NA	NE	NA	NA	NA
CUP#126	Negative	55	Lymph node	Female	No	Yes	*HBV + HCV - HPV NA*	Carcinoma	1	Lymph nodes	1	F	Folfox	NA	PD
CUP#127	Negative	82	Bone	Male	Yes	No	*HBV - HCV - HPV NA*	Adenocarcinoma	1	Bone	1	U	NA	NA	NA
CUP#128	First-degree relative: undefined cancer	81	Lymph node	Female	No	Yes	*HBV - HCV - HPV NA*	Carcinoma	1	Lymph nodes	1	F	Carboplatin + Taxol	Yes	PR
CUP#129	First-degree relative: breast and gallbladder cancer; other first-degree relative: lung and pancreas cancer	71	Soft tissuses	Female	Yes	No	*HBV - HCV - HPV NA*	Carcinoma	1	Soft tissue	0	U	Carboplatin + Gemcitabine	Yes	PD
CUP#130	Unknown	69	Soft tissus	Female	Yes	No	*HBV - HCV - HPV NA*	Carcinoma	2	Lymph nodes, soft tissue	0	U	Xelox	NA	PR
CUP#131	Unknown	65	Lymph node	Male	Yes	Yes	*HBV - HCV - HPV NA*	Carcinoma	3	Lymph nodes, liver, bone	1	U	Carboplatin + Taxol	NA	PD
CUP#132	First-degree relative: unknow cancer	81	Bone	Male	No	Yes	*HBV - HCV - HPV NA*	Carcinoma	1	Bone	1	U	NO	NA	NA
CUP#133	First-degree relative: stomach cancer; first-degree relative: unknow cancer; other first-degree relative: lung cancer; second-degree relative: unknow cancer	70	Bone	Male	Yes	No	*HBV - HCV - HPV NA*	Adenocarcinoma	2	Bone, lymph nodes	3	U	NO	NA	NA
CUP#134	Negative	51	Mesentery	Male	No	No	*HBV + HCV - HPV +*	Adenocarcinoma	6	Lung, lymph nodes, liver, adrenal gland dx, peritoneum, bone	0	U	Folfox	NA	PD
CUP#137	First-degree relative: lung cancer	73	Lymph node	Female	Yes	No	*HBV - HCV - HPV NA*	Leiomyosarcoma	3	Liver, lung, bone	0	U	Doxorubicina	NA	PD
CUP#138	Second-degree relative: HCC; other second-degree relative: ovary cancer	32	Ovaries	Female	NA	NA	*HBV + HCV - HPV NA*	Carcinoma	2	Pelvis, bone	3	U	De Gramont + Zometa	NA	PD
CUP#139	Negative	68	Lymph node	Male	Yes	No	*HBV - HCV - HPV NA*	Carcinoma	1	Lymph nodes	0	F	Pembrolizumab + Axitinib	NA	PR
CUP#140	Positive	42	Liver	Male	NA	NA	*HBV NA HCV NA HPV NA*	Adenocarcinoma	2	Liver, lymph nodes	1	U	Cisplatin + Gemcitabine	NA	SD
CUP#141	Unknown	74	NA	Male	NA	NA	*HBV NA HCV NA HPV NA*	NA	NA	NA	NA	NE	NA	NA	NA
CUP#142	Unknown	69	Lymph node	Male	NA	NA	*HBV NA HCV NA HPV NA*	Adenocarcinoma	NA	NA	NA	NE	NA	NA	NA
CUP#143	First-degree relative:colon cancer	75	Bone	Female	No	No	*HBV - HCV - HPV NA*	Carcinoma	2	Bone, lymph nodes	1	U	Carboplatin + Taxol	NA	SD
CUP#154	Unknown	84	Liver	Female	No	No	*HBV - HCV - HPV NA*	Adenocarcinoma	3	Liver, lung, lymph nodes	2	U	NO	NA	NA
CUP#155	First-degree relative: rectum cancer; first-degree relative: stomach cancer	65	Lymph node	Female	Yes	Yes	*HBV - HCV - HPV NA*	Adenocarcinoma	1	Lymph nodes	2	F	Folfox	NA	PD
CUP#156	First-degree relative:breast cancer; second-degree relative: ovary cancer; other second-degree relative: prostate cancer	53	Liver	Female	No	No	*HBV - HCV - HPV NA*	Carcinoma	4	Ovary, liver, peritoneum, intestine	0	U	Folfox	NA	NA
CUP#157	Negative	56	Peritoneum	Female	No	Yes	*HBV - HCV - HPV NA*	Carcinoma	3	Intestine, peritoneum, ovary	0	U	Folfox	NA	PD
CUP#162	Negative	46	Lung	Female	No	No	*HBV - HCV - HPV NA*	Carcinoma	1	Pleura	0	U	Carboplatin + Pemetrexed + Pembrolizumab	NA	PR
CUP#163	First-degree relative: lung cancer	56	Liver	Female	No	No	*HBV - HCV - HPV +*	NET	1	Liver	0	F	Captem	NA	PR
CUP#164	First-degree relative: breast cancer; first-degree relative: unknown cancer	71	Ovaries	Female	No	No	*HBV - HCV - HPV NA*	Adenocarcinoma	2	Ovary, peritoneum	0	U	Xelox	NA	PR
CUP#165	First-degree relative: unknown cancer; first-degree relative: unknown cancer	56	Lymph node	Female	No	No	*HBV - HCV + HPV +*	Carcinoma	2	Lymph nodes, liver	1	U	Cisplatin + 5-FU + Pembrolizumab	Yes	PR
CUP#166	Unknown	81	Bone	Male	NA	NA	*HBV NA HCV NA HPV NA*	Adenocarcinoma	NA	NA	NA	NE	NA	NA	NA

*U, unfavorable group; F, favorable group. NA, not available; NE, not evaluable. NIH definition of degree relatives: A first-degree relative is a family member who shares about half of their genetic information with specific other individuals in their family. First-degree relatives include individual’s parents, siblings and offspring. Second degree relatives: An aunt, uncle, grandparent, grandchild, niece, nephew, or half-sibling of an individual. Also called SDR. Third-degree relatives: A first cousin, great-grandparent, great-aunt, great-uncle, great-niece, great-nephew, great-grandchild, half-aunt, or half-uncle of an individual. Also called TDR.

A comprehensive review of patients’ medical records was performed to systematically collect clinicopathological data. Collected variables included demographic, clinical, and molecular characteristics. Specifically, age at diagnosis, Eastern Cooperative Oncology Group (ECOG) performance status, sex, and lifestyle factors such as smoking and alcohol consumption were recorded. Infection status, including HIV, HBV, HCV, and HPV, was also documented.

Tumor characterization included histological subtype, immunohistochemical (IHC) analyses, and molecular profiling. Diagnostic next-generation sequencing (NGS) was performed on formalin-fixed paraffin-embedded (FFPE) tissue samples using the Oncomine Focus Assay (Thermo Fisher Scientific, Carlsbad, CA, USA; research-use-only [RUO] kit). Data on disease progression, including the number and sites of metastases and biopsy site, were collected. Treatment history was reviewed to document the number and types of systemic therapies administered.

A total of 41 ccfDNA samples were collected from 39 CUP patients, including longitudinal samples obtained at diagnosis and disease progression from two patients (CUP#55 and CUP#128; samples a and c, respectively). Germline genomic DNA (gDNA) was available for 32 patients and was derived from whole blood (*n* = 1), peripheral blood mononuclear cells (PBMCs; *n* = 29), and FFPE normal tissue (*n* = 2). For seven patients (CUP#009, CUP#051, CUP#052, CUP#054, CUP#137, CUP#138, CUP#143), matched genomic DNA was not available.

### Blood collection and DNA isolation

Peripheral blood samples (10 mL) were collected in EDTA Vacutainer tubes. All samples were processed within 3 h of collection. Plasma was obtained by centrifugation at 1900 × g for 10 min at 4 °C and stored at −80 °C in 1 mL aliquots until use.

Cell-free DNA (cfDNA) was extracted from 1 mL of plasma using the Maxwell RSC instrument (Cat No: AS4500, Promega, Madison, WI, USA) with the Maxwell RSC ccfDNA Plasma Kit (Cat No: AS1480, Promega), according to the manufacturer’s instructions. Samples were assessed for genomic DNA (gDNA) contamination by loading 1 μL of cfDNA onto an Agilent Genomic DNA ScreenTape (Cat No: 5067–5,365) using the TapeStation system. Samples were considered free of gDNA contamination when they exhibited the characteristic cfDNA fragmentation pattern in the absence of a high-molecular-weight DNA smear (>1,000 bp). Only samples meeting these criteria were included in downstream NGS analysis.

Genomic DNA (gDNA) from PBMCs (*n* = 30) was extracted using the Maxwell CSC Blood DNA Kit (Cat No: AS1321), following the manufacturer’s instructions. cfDNA was quantified using the Qubit 4.0 Fluorometer (Cat No: Q33238, Thermo Fisher Scientific, Waltham, MA, USA). For patients CUP#33 and CUP#95, gDNA was extracted from non-tumoral areas of FFPE tissue due to the unavailability of PBMCs. FFPE samples were processed as previously described (15). DNA extraction was performed using the QIAamp DNA FFPE Tissue Kit (Cat No: 56404, Qiagen, Hilden, Germany), according to the manufacturer’s instructions. gDNA concentration was measured using the NanoDrop One/OneC Microvolume UV–Vis Spectrophotometer (Thermo Fisher Scientific).

### Circulating tumor DNA (ctDNA) quantification

The ctDNA fraction within ccfDNA was calculated as previously described (24), by multiplying the maximum validated somatic variant allele frequency (VAF) by the total ccfDNA concentration obtained from 1 mL of plasma (ng/mL):


$$\begin{array}{l} \text{ctDNA concentration}\;(\mathrm{ng}/\mathrm{mL})=\text{maximum}\;\mathrm{VAF}\\ \times \text{ccfDNA concentration}\;(\mathrm{ng}/\mathrm{mL}). \end{array}$$

### Next, generation sequencing

#### Library generation

A custom panel targeting 92 cancer-related genes most frequently mutated in CUP patients, based on the GENIE project (25) and MSK-IMPACT studies (26), was designed using the Agilent SureSelect XT platform, as previously described (27). Briefly, the Agilent SureDesign web application (v7.0) was used to design the panel, which included all coding exons, untranslated regions (UTRs), and the first 25 nucleotides of intronic sequences at both the 3′ and 5′ ends. The panel covered a total genomic region of 1.2 Mb (Agilent Technologies, Santa Clara, CA, USA). A total of 73 libraries were generated using the SureSelectXT HS/SureSelectXT custom low-input library kit, following the manufacturer’s Target Enrichment protocol (G9702-90005, v. A0, June 2019). Input DNA consisted of 25 ng of ccfDNA or 100 ng of PBMC- or FFPE-derived gDNA. Libraries were quantified using D1000 ScreenTape reagents (Agilent Technologies), the 4,150 TapeStation system (G2992AA), and a Qubit fluorometer (Thermo Fisher Scientific). Libraries were normalized to 4 nM and pooled in equimolar amounts. Multiplexed libraries were quantified using Agilent High Sensitivity D1000 reagents and sequenced on a NextSeq 500 platform (Illumina) using High Output 2 × 75 bp flow cells.

#### Sequence alignment and quality control

##### FASTQ file generation

Raw BCL (Binary Base Call) files were processed using bcl2fastq v2.19.0.316 to generate FASTQ files. The base-mask option was applied to generate dedicated molecular barcode FASTQ files. Sequencing reads were aligned to the human reference genome (GRCh38), and UMI collapsing, variant calling, annotation, and downstream analyses were performed using Agilent SureCall v4.2.2.3.

##### Variant calling

Variants were identified using the SNPPET SNP caller implemented in Agilent SureCall v4.2.2.3, applying the following criteria: variant score threshold ≥0.3, minimum base quality ≥30, variant call quality threshold ≥100, and minimum allele frequency of 1%. Variant Calling Format (VCF) files were generated to identify sequence variations relative to the reference genome. Internal quality control (QC) parameters implemented in SureCall were used to distinguish true variants from potential artifacts arising during library preparation, target enrichment, sequencing, or alignment. The average sequencing coverage (read depth) was 612 (range 179–1933) for ccfDNA samples, 493 (range 224–1,086) for PBMC samples, and 18 (range 12–33) for normal FFPE samples. Normal FFPE samples were used exclusively for germline comparison purposes.

### Cancer variant identification, annotation, and interpretation

#### Computational tools and annotation databases

All variants identified in VCF files were annotated and functionally interpreted using ANNOVAR (28) and the Open Custom Ranked Analysis of Variants Toolkit (OPENCRAVAT, v2.2.7). The clinical and biological relevance of somatic variants was further assessed using the OncoKB knowledge base (29), accessed through the OncoKB Annotator.^1^

Variants were converted from VCF to MAF format using vcf2maf (Kandoth C. mskcc/vcf2maf: vcf2maf v1.6. 2020, doi:10.5281/zenodo.593251) to enable standardized downstream analyses. MAF files were subsequently analyzed using the maftools package for summarization and visualization of somatic mutation profiles. Graphical representations were generated using ggplot2, while co-mutation patterns were visualized with CoMut (30). Pathway-level representations of altered genes were generated using PathwayMapper (31).

Annotation with ANNOVAR was performed using databases retrieved from the UCSC Genome Browser Annotation Database, including refGene, avsnp150, ljb26_all, cosmic70, ClinVar (release 20,220,320), InterVar (20180118), and gnomAD (v2.1.1 exome). OPENCRAVAT annotation was performed using an extensive set of modules, including AlphaMissense, BioGRID, Cancer Genome Interpreter, Cancer Hotspots, CIViC, ClinGen, ClinVar, COSMIC, gnomAD, OMIM, PharmGKB, UniProt, and additional functional and population-based resources.

Outputs from ANNOVAR, OPENCRAVAT, and OncoKB were integrated into a unified dataset using a custom R-based pipeline, including harmonization of gene symbols, variant representations, functional annotations, and clinical classifications. This integrated dataset provided a comprehensive multi-dimensional annotation framework and was used for downstream variant filtering, prioritization, and classification.

#### Variant filtering and prioritization

##### Variant prioritization and classification

Only exonic variants were included in the analysis, while variants located in 3′ UTR regions and exon junction boundaries were excluded. Among exonic variants, synonymous single nucleotide variants (SNVs) were removed. Variants were classified based on variant allele frequency (VAF), as described below. Common variants with a minor allele frequency (MAF) > 1% in the Genome Aggregation Database (gnomAD), which aggregates genomic data from over 140,000 individuals across multiple populations (32), were excluded.

##### Identification and interpretation of germline variants

Putative germline variants were defined as variants detected in both ccfDNA and matched gDNA after excluding common population variants (MAF > 1% in gnomAD) and synonymous SNVs. Variants were classified as heterozygous if the VAF ranged from 40 to 60% and as homozygous if the VAF exceeded 95%. In patients lacking matched gDNA samples, germline variants were inferred based on VAF thresholds in ccfDNA (40–60% for heterozygous variants and >95% for homozygous variants) (33). However, in unmatched cases, somatic variants with high allele frequency, particularly in samples with elevated ctDNA fraction, may be misclassified as germline, although this risk is considered low (34). Germline variant pathogenicity was assessed according to ACMG/AMP guidelines (35), incorporating ClinGen Sequence Variant Interpretation (SVI) recommendations. When available, gene-specific ClinGen Variant Curation Expert Panel (VCEP) specifications were applied. Computational predictions from AlphaMissense were used as supporting evidence (ACMG PP3/BP4) and interpreted in conjunction with population, clinical, and functional data.

##### Identification and interpretation of somatic variants

In paired samples (*n* = 34), somatic variants were identified based on their presence in ccfDNA and absence in matched gDNA derived from PBMCs or normal FFPE tissue. This paired approach enabled reliable discrimination of somatic variants. For the seven samples lacking matched gDNA, somatic variants were defined as those with VAF between 1 and 40% or between 60 and 95%, after excluding variants with MAF > 1% in gnomAD. Somatic variants (Supplementary Table 1) were annotated using the OncoKB knowledge base according to established guidelines for somatic variant interpretation and oncogenicity assessment (36, 37). For each variant, the highest level of evidence was retrieved considering gene, alteration, and tumor context. Clinical significance was classified according to OncoKB levels of evidence (Levels 1–4), which were mapped to the AMP/ASCO/CAP tier system: Levels 1–2 correspond to Tier I, Levels 3A–3B to Tier II, and Level 4 to Tier II–III, irrespective of tumor type. Oncogenicity was assessed using OncoKB variant-level classifications (oncogenic, likely oncogenic, likely neutral, or inconclusive). Variants were reported in Table 2 if classified as Tier I–III, and their oncogenic or likely oncogenic status was indicated. All remaining variants are reported in the Supplementary material.

**Table 2:** Clinically relevant somatic mutations detected in CUP ccfDNA.

Patient	Gene	Genetic alteration	VAF	Detected in tumor tissue DNA (method)*	Mutation_effect (OncoKB)	Oncogenic (OncoKB)	OncoKB levels of evidence	Highest level therapies	Tier
CUP#033	NF1	p.K191Nfs*10	2.65%	Inadequate	Likely Loss-of-function	Likely Oncogenic	L1, L4	Mirdametinib, Selumetinib	TierI
CUP#051	BRAF	p.D594G	8.62%	Inadequate	Gain-of-function	Oncogenic	L2, L3A	Cobimetinib, Trametinib	TierI
CUP#052	KIT	p.L576P	19.79%	p.L576P (exon 11 Sanger sequencing)	Gain-of-function	Oncogenic	L1, L2	Imatinib, Sunitinib, Regorafenib, Ripretinib	TierI
	NF1	p.Q803*	9.44%	NA	Likely Loss-of-function	Likely Oncogenic	L1, L4	Mirdametinib, Selumetinib	TierI
	NF1	p.N39Tfs*5	12.32%	NA	Likely Loss-of-function	Likely Oncogenic	L1, L4	Mirdametinib, Selumetinib	TierI
CUP#055a	ARID1A	p.R1276*	25.16%	p.R1276* (NGS Roche Foundation One)	Likely Loss-of-function	Likely Oncogenic	L4	OPN-2853, Tazemetostat	Tier II–III
CUP#055c	ARID1A	p.R1276*	25.26%	p.R1276* (Roche Foundation One)	Likely Loss-of-function	Likely Oncogenic	L4	OPN-2853, Tazemetostat	Tier II–III
	ATR	p.I774Yfs*5	1.09%	NA	Likely Loss-of-function	Likely Oncogenic	L1	Talazoparib+Enzalutamide	TierI
CUP#062	NF1	p.K191Nfs*10	3.58%	NA	Likely Loss-of-function	Likely Oncogenic	L1, L4	Mirdametinib, Selumetinib	TierI
CUP#063	NF1	p.K191Nfs*10	4.70%	NA	Likely Loss-of-function	Likely Oncogenic	L1, L4	Mirdametinib, Selumetinib	TierI
CUP#064	NF1	p.K191Nfs*10	6.52%	NA	Likely Loss-of-function	Likely Oncogenic	L1, L4	Mirdametinib, Selumetinib	TierI
CUP#090	ARID1A	p.I1485Rfs*19	38.92%	NA	Likely Loss-of-function	Likely Oncogenic	L4	OPN-2853, Tazemetostat	Tier II–III
CUP#095	KRAS	p.G12C	39.44%	p.G12C (NGS Oncomine Focus Assay)	Gain-of-function	Oncogenic	L1, L2, L3A, L4, LR1	Adagrasib, Adagrasib+Cetuximab, Avutometinib+Defactinib, Sotorasib, Sotorasib+Panitumumab	TierI
	NF1	p.K191Nfs*10	2.56%	NA	Likely Loss-of-function	Likely Oncogenic	L1, L4	Mirdametinib, Selumetinib	TierI
	PIK3CA	p.K111E	2.23%	Not detected	Gain-of-function	Oncogenic	L1, L2, L4	Inavolisib+Palbociclib+Fulvestrant	TierI
CUP#124	NF1	p.K191Nfs*10	3.33%	Inadequate	Likely Loss-of-function	Likely Oncogenic	L1, L4	Mirdametinib, Selumetinib	TierI
CUP#125§	ATR	p.I774Nfs*3	1.13%	NA	Likely Loss-of-function	Likely Oncogenic	L1	Talazoparib+Enzalutamide	TierI
	KRAS	p.G12C	1.92%	KRAS G12C (NGS Oncomine Focus Assay)	Gain-of-function	Oncogenic	L1, L2, L3A, L4, LR1	Adagrasib, Adagrasib+Cetuximab, Avutometinib+Defactinib, Sotorasib, Sotorasib+Panitumumab	TierI
	STK11	p.R297S	2.67%	NA	Likely Loss-of-function	Likely Oncogenic	L4	Bemcentinib+Pembrolizumab	Tier II–III
CUP#126	ARID1A	p.D1850Gfs*4	5.96%	NA	Likely Loss-of-function	Likely Oncogenic	L4	OPN-2853, Tazemetostat	Tier II–III
	ATM	p.K2811Sfs*46	3.59%	NA	Likely Loss-of-function	Likely Oncogenic	L1	Olaparib, Talazoparib+Enzalutamide	TierI
	ATR	p.I774Yfs*5	6.93%	NA	Likely Loss-of-function	Likely Oncogenic	L1	Talazoparib+Enzalutamide	TierI
	CDKN2A	p.R80*	5.45%	NA	Likely Loss-of-function	Likely Oncogenic	L4	Palbociclib, Ribociclib, Abemaciclib	Tier II–III
	CDKN2A	p.R58*	6.57%	NA	Likely Loss-of-function	Likely Oncogenic	L4	Palbociclib, Ribociclib, Abemaciclib	Tier II–III
	KRAS	p.Q61H	13.06%	NA	Gain-of-function	Oncogenic	L1, L2, L3A, L4, LR1	Avutometinib+Defactinib	TierI
	NF1	p.K191Nfs*10	3.77%	NA	Likely Loss-of-function	Likely Oncogenic	L1, L4	Mirdametinib, Selumetinib	TierI
CUP#129	NF1	p.C851*	7.44%	NA	Likely Loss-of-function	Likely Oncogenic	L1, L4	Mirdametinib, Selumetinib	TierI
	PALB2	p.G999*	2.21%	NA	Likely Loss-of-function	Likely Oncogenic	L1, L2	Olaparib, Talazoparib+Enzalutamide	TierI
	PIK3CA	p.E545K	8.92%	p.E545K (NGS Oncomine Focus Assay)	Gain-of-function	Oncogenic	L1, L4	Alpelisib+Fulvestrant, Capivasertib+Fulvestrant, Ivolisib+Palbociclib+Fulvestrant	TierI
CUP#131	ATM	p.L1814Wfs*14	1.66%	NA	Likely Loss-of-function	Likely Oncogenic	L1,	Olaparib, Talazoparib+Enzalutamide	TierI
	FBXW7	p.D705Efs*3	2.49%	NA	Likely Loss-of-function	Likely Oncogenic	L3A, L4	Lunresertib+Camonsertib	TierII
CUP#133	ARID1A	p.R1276*	7.32%	NA	Likely Loss-of-function	Likely Oncogenic	L4	OPN-2853, Tazemetostat	Tier II–III
	FBXW7	p.R222*	9.08%	NA	Likely Loss-of-function	Likely Oncogenic	L3A, L4	Lunresertib+Camonsertib	TierII
CUP#134	ARID1A	p.D1850Tfs*33	3.70%	NA	Likely Loss-of-function	Likely Oncogenic	L4	OPN-2853, Tazemetostat	Tier II–III
	KRAS	p.G12V	8.01%	p.G12V (NGS Oncomine Focus Assay)	Gain-of-function	Oncogenic	L1, L2, L3A, L4, LR1	Avutometinib+Defactinib	TierI
CUP#143	ERBB2	p.L755S	7.69%	Inadequate	Gain-of-function	Oncogenic	L1, L2, L3A, L4	Sevabertinib, Trastuzumab Deruxtecan, Zongertinib	TierI
	PIK3CA	p.G118D	7.86%	Inadequate	Gain-of-function	Oncogenic	L1, L2, L4	Inavolisib+Palbociclib+Fulvestrant	TierI
CUP#154	ARID1A	p.E1444*	25.22%	NA	Likely Loss-of-function	Likely Oncogenic	L4	OPN-2853, Tazemetostat	Tier II–III
CUP#156	KRAS	p.G12D	4.22%	p.G12D (NGS Oncomine Focus Assay)	Gain-of-function	Oncogenic	L1, L2, L3A, L4, LR1	Avutometinib+Defactinib	TierI
CUP#157	ERBB2	p.S310F	3.38%	p.S310F (NGS Oncomine Focus Assay)	Gain-of-function	Oncogenic	L1, L2, L3A, L4	Trastuzumab Deruxtecan	TierI
	FBXW7	p.R465H	2.90%	NA	Loss-of-function	Likely Oncogenic	L3A, L4	Lunresertib+Camonsertib	TierII
	KRAS	p.G12D	5.86%	p.G12D (NGS Oncomine Focus Assay)	Gain-of-function	Oncogenic	L1, L2, L3A, L4, LR1	Avutometinib+Defactinib	TierI
CUP#165	PIK3CA	p.E545K	16.67%	p.E545K (NGS Oncomine Focus Assay)	Gain-of-function	Oncogenic	L1, L4	Alpelisib+Fulvestrant, Capivasertib+Fulvestrant, Inavolisib+Palbociclib+Fulvestrant	TierI
CUP#166	KRAS	p.G12V	12.52%	p.G12V (NGS Oncomine Focus Assay)	Gain-of-function	Oncogenic	L1, L2, L3A, L4, LR1	Avutometinib+Defactinib	TierI

^*^NA, the gene was not available in the gene panel used for diagnostics or the tumor tissue DNA was not tested. Inadequate, the DNA from tumor tissue was inadequate for molecular diagnostic assessment. Not detected, the mutation was not detected in tumor tissue DNA molecular diagnostic assessment. ^§^CUP#125 has CTNNB1 p.D32V mutation in tumor tissue (NGS Oncomine Focus Assay).

##### Clonal hematopoiesis of indeterminate potential (CHIP)

Genes associated with clonal hematopoiesis (38, 39) included *ASXL2, BCOR, BRAF, CREBBP, EP300, JAK3, KIT, KRAS, NF1, NOTCH1, NOTCH2, NRAS, SETD2, STAG2, and TP53*. Variants detected in these genes in PBMC-derived gDNA with VAF between 2 and 35% were classified as CHIP-related mutations, consistent with clonal expansion of mutated hematopoietic cells (22).

### Statistical analysis

Statistical analyses were performed using R software. Survival analyses were conducted using the survival package (v3.8–3) (Therneau TM. A Package for Survival Analysis in R [Internet]. 2020. Available from: https://CRAN.R-project.org/package=survival). Overall survival (OS) and progression-free survival (PFS) were estimated using the Kaplan–Meier method and compared between groups using the log-rank (Mantel–Cox) test. Patients were dichotomized based on circulating cell-free DNA (ccfDNA) concentration and circulating tumor DNA (ctDNA) fraction. Optimal cut-off values were determined using a receiver operating characteristic (ROC) curve–based approach, selecting the threshold that maximized the area under the curve (AUC). All statistical tests were two-sided, and a *p*-value < 0.05 was considered statistically significant.

## Results

### Patient characteristics, real-world clinical management, and outcomes

Thirty-nine patients diagnosed with cancer of unknown primary (CUP) were enrolled in this study. Patient demographics and baseline characteristics are summarized in Table 1. None of the patients had a history of prior malignancies or had received chemotherapy before enrollment. All patients presented with at least one metastatic lesion, and no primary tumor could be identified at diagnosis. Biopsy and immunohistochemical analyses were performed on a metastatic site.

The most common histological subtype was carcinoma (*N* = 34), including 11 adenocarcinomas. The cohort comprised 24 females and 15 males, with a mean age at diagnosis of 65.5 years (range, 32–87).

We evaluated the prevalence of previously reported CUP risk factors. Among patients with available data, 14/32 reported a history of smoking, 12/32 reported alcohol consumption, and 21/29 had a family history of cancer. Seropositivity for hepatitis B virus (HBV) and hepatitis C virus (HCV) was observed in 4/35 and 1/35 patients, respectively; one patient was co-infected with both viruses. In addition, three patients tested positive for HPV based on p16 immunostaining.

The number of metastatic sites per patient ranged from one to six. The most frequent metastatic sites included lymph nodes, peritoneum, liver, lung, pleura, intestine, soft tissue, bone, and brain. ECOG performance status ranged from 0 to 3: among 35 evaluable patients, 12 had ECOG 0, 15 had ECOG 1, and 8 had ECOG ≥2; ECOG data were not available for four patients. According to ESMO clinical guidelines, patients were classified into favorable (*N* = 6) and unfavorable (*N* = 29) prognostic groups.

Chemotherapy was administered to 30 patients, while three did not receive chemotherapy; treatment data were unavailable for six patients. In a subset of patients, treatment decisions were guided by clinicopathological features suggestive of a potential tissue of origin. For example, patients with gastrointestinal-like profiles were treated with fluoropyrimidine-based regimens (e.g., FOLFOX or XELOX), while selected cases received tumor-specific therapies based on suspected origin (e.g., renal, lung, head and neck, or breast-like profiles). Most patients received platinum-based regimens combined with gemcitabine (*N* = 5), paclitaxel (*N* = 4), pemetrexed (*N* = 1), or 5-fluorouracil (*N* = 2). Additional regimens included anthracycline-based chemotherapy (*N* = 2), CAPTEM (*N* = 1), GEMOX (*N* = 1), and zoledronic acid (*N* = 1). Two patients received tyrosine kinase inhibitors, two received immunotherapy, and one was treated with cetuximab. Additional regimens included anthracycline-based chemotherapy (*N* = 2), CAPTEM (*N* = 1), GEMOX (*N* = 1), and zoledronic acid (*N* = 1). Two patients received tyrosine kinase inhibitors, two received immunotherapy, and one was treated with cetuximab. Among patients who received second-line therapy, two patients (CUP#128 and CUP#129) were treated with immunotherapy based on PD-L1 positivity. For the remaining patients, treatment decisions were not guided by genomic profiling.

Best treatment response was available for 29 patients and included nine partial responses, six cases of stable disease, and 14 cases of progressive disease. Ten patients received second-line therapy.

#### Somatic variant identification in CUP ctDNA and functional classification

**Figure 1:**
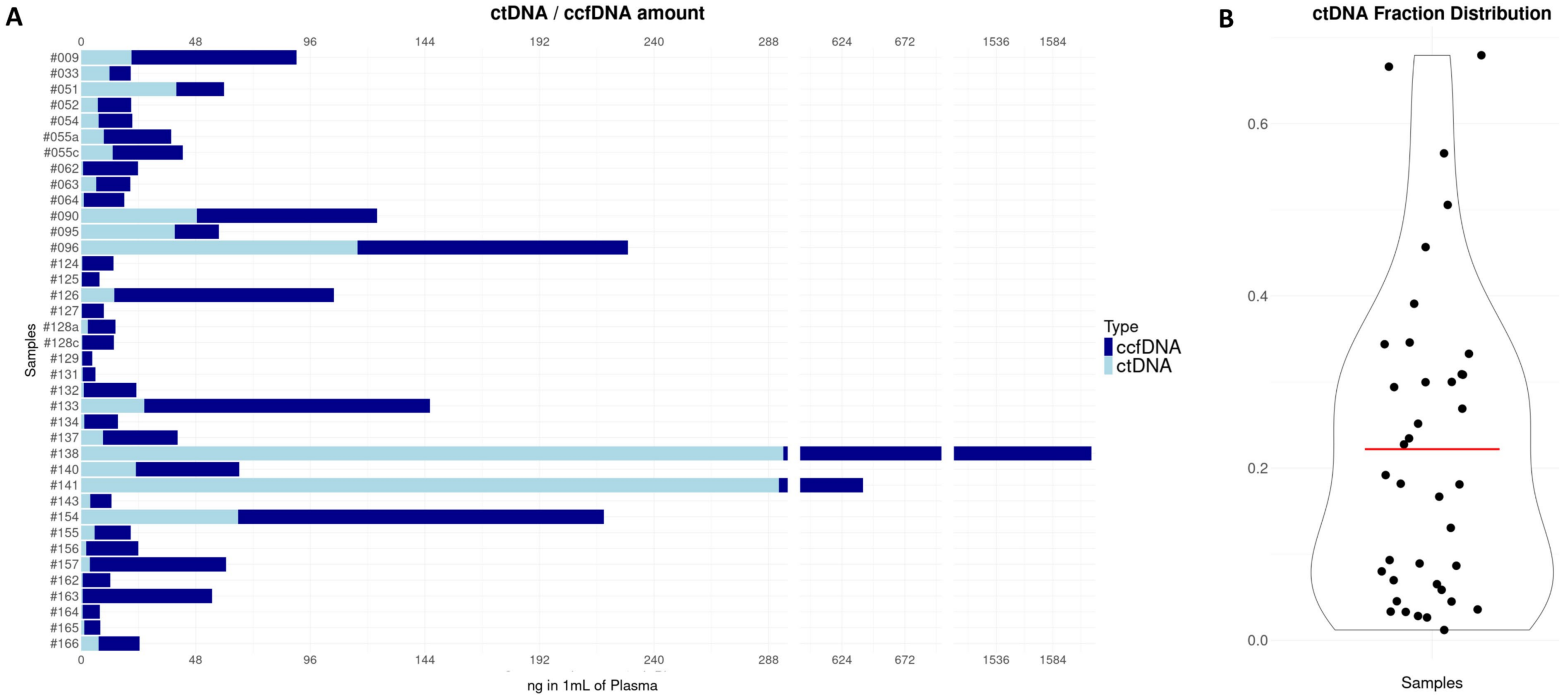
Quantification of ctDNA in CUP patients. **(A)** Histogram showing circulating cell-free DNA (ccfDNA) and circulating tumor DNA (ctDNA) levels in 41 plasma samples, expressed as total DNA amount per 1 mL of plasma (x-axis). The ctDNA fraction was estimated using the maximum variant allele frequency (VAF) detected in each sample. Dark blue bars represent total ccfDNA, while light blue bars indicate the estimated ctDNA fraction. **(B)** Violin plot illustrating the distribution of ctDNA fraction across the 41 ccfDNA samples. The y-axis represents the ctDNA fraction.

A total of 41 ccfDNA samples were collected from 39 CUP patients, including longitudinal samples obtained at diagnosis and disease progression from two patients (CUP#55 and CUP#128; samples a and c, respectively). Plasma ccfDNA concentrations ranged from 6.2 ng/mL to >2 μg/mL (Figure 1A), indicating markedly elevated levels. Targeted sequencing was performed on 73 samples, comprising ccfDNA from 39 patients and matched gDNA from 32 patients, using a CUP-specific 92-gene panel.

After variant filtering and prioritization, exonic, non-synonymous variants with a minor allele frequency <1% in gnomAD were analyzed independently of their predicted oncogenicity to characterize the mutational landscape of CUP. Somatic mutation profiles for all patients, including longitudinal samples, are provided in Supplementary Table 1. The number of mutated genes per patient ranged from 0 to 24. Three patients (CUP#130, CUP#139, and CUP#142) did not harbor detectable somatic mutations in the genes analyzed. The ctDNA fraction, estimated using the maximum variant allele frequency (VAF), reached up to 67% of total ccfDNA in individual samples (Figure 1B).

**Figure 2:**
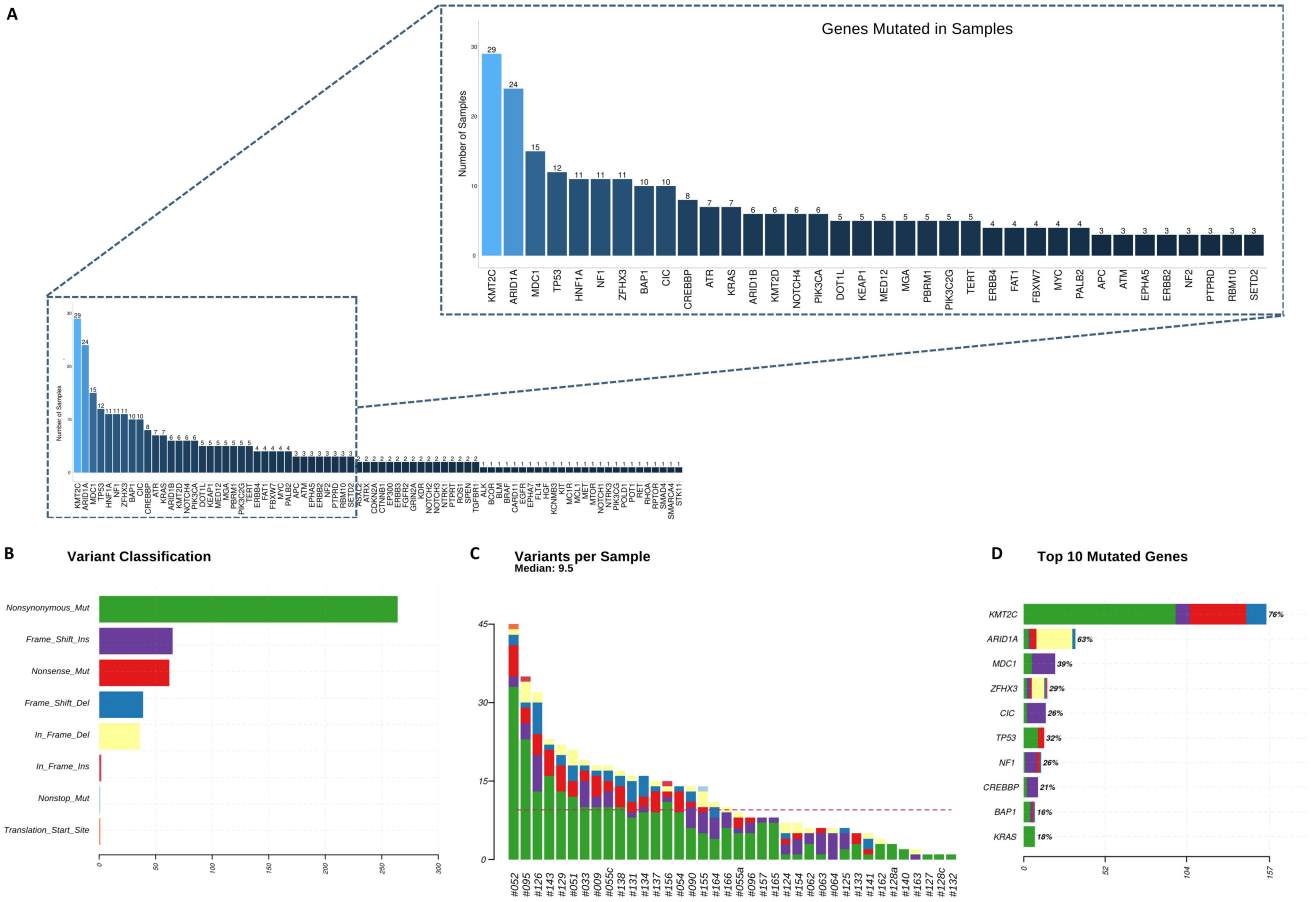
Somatic mutation landscape and variant classification. **(A)** Distribution of genes harboring somatic mutations identified by targeted sequencing of 41 CUP samples using a custom 92-gene panel. The number above each bar indicates the number of samples with at least one mutation in the corresponding gene. Genes mutated in ≥3 patients are highlighted in the inset. **(B)** Functional classification of somatic variants according to variant type (missense, nonsense, frameshift insertion/deletion, in-frame deletion). **(C)** Number of somatic variants detected per sample. Each bar represents an individual CUP sample. The horizontal red dashed line indicates the median number of variants per sample (9.5). **(D)** Top 10 most frequently mutated genes. Bar length indicates the total number of variants per gene, and the percentage of mutated samples is shown on the right. Variant type color coding is consistent with panel **(B)**.

Somatic mutations were identified in 78 genes, corresponding to the majority of genes included in the panel, with 36 of 39 patients harboring at least one somatic mutation (Figure 2A). The most frequently mutated genes included *KMT2C, ARID1A, MDC1, TP53, HNF1A, NF1, ZFHX3, BAP1, and CIC*. Overall, 36 genes were mutated in at least three patients (Figure 2A).

Missense mutations represented the most common mutation class, followed by frameshift insertions and nonsense mutations (Figure 2B). Analysis of nucleotide substitution patterns revealed a predominance of C>T transitions, accounting for more than 50% of detected mutations (Supplementary Table 1). On average, patients harbored 9.5 somatic mutations in ctDNA. Patients CUP#052, CUP#095, and CUP#126 exhibited the highest mutational burden, with more than 30 mutations each (Figure 2C), consistent with CUP#126 being MSH2-negative and therefore MSI-H according to clinical records. The distribution of mutation types among the top 10 mutated genes is shown in Figure 2D.

Somatic mutations were annotated using the OncoKB precision oncology knowledge base to assess oncogenicity and clinical significance. Mutations were classified according to their biological effect and assigned levels of evidence, which were subsequently mapped to AMP/ASCO/CAP tiers to define their potential therapeutic, diagnostic, and prognostic relevance across tumor types. Only mutations with clinical significance corresponding to AMP/ASCO/CAP Tiers I–III were included in Table 2, whereas all annotated variants are reported in Supplementary Table 1. Oncogenicity of mutations included in Table 2 was defined based on OncoKB classifications, with AlphaMissense predictions provided as supportive evidence. Concordance with mutation detection in tumor tissue biopsy, based on available clinical records, was also reported.

#### Recurrent somatic alterations and pathway classification

**Figure 3:**
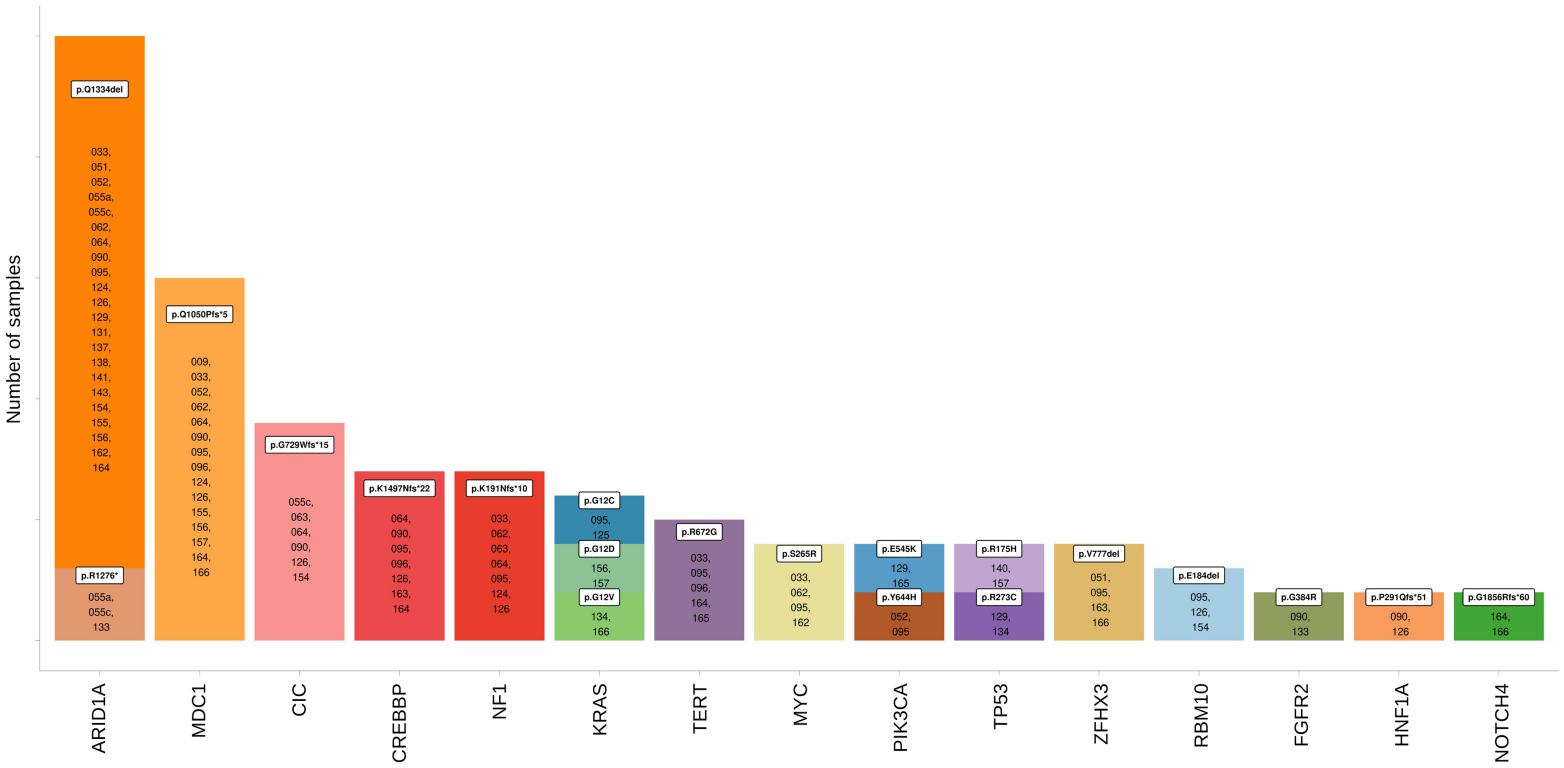
Recurrent somatic genetic alterations. Bar plot illustrating specific recurrent somatic genetic alterations shared by at least two CUP patients. Genes are shown on the x-axis. Each box represents a distinct mutation, and the CUP samples harboring that mutation are reported within each box. Bar height (y-axis) is proportional to the number of samples.

Several recurrent exonic, non-synonymous somatic mutations with a minor allele frequency <1% in gnomAD were shared among multiple patients (Figure 3; Supplementary Table 2), suggesting a role for these alterations in the early acquisition of the aggressive and metastatic phenotype characteristic of CUP, independently of the tissue of origin.

The most frequent alteration was *ARID1A* Q1334del, detected in 21 of 39 patients (55%). This variant consists of a three-nucleotide deletion within a homopolymeric region and therefore requires cautious interpretation; however, it has been reported in CUP cases from the AACR GENIE dataset. The *MDC1* Q1050Pfs5 frameshift mutation was detected in 15 patients (39%). Frameshift mutations in *CREBBP* (K1497Nfs22) and *NF1* (K191Nfs10) were each identified in 18% of patients, while *CIC* G729Wfs15 occurred in six patients (15%). Activating *KRAS* codon 12 mutations (G12C/D/V) were detected in six patients, and *TERT* R672G was observed in five patients (13%).

Due to their high mutation frequency and complex mutational pattern, *KMT2C* alterations (*N* = 26) were excluded from Figure 3 for clarity and are reported in Supplementary Table 2. Notably, patient CUP#64 harbored multiple independent frameshift mutations, suggestive of possible DNA mismatch repair deficiency.

**Figure 4:**
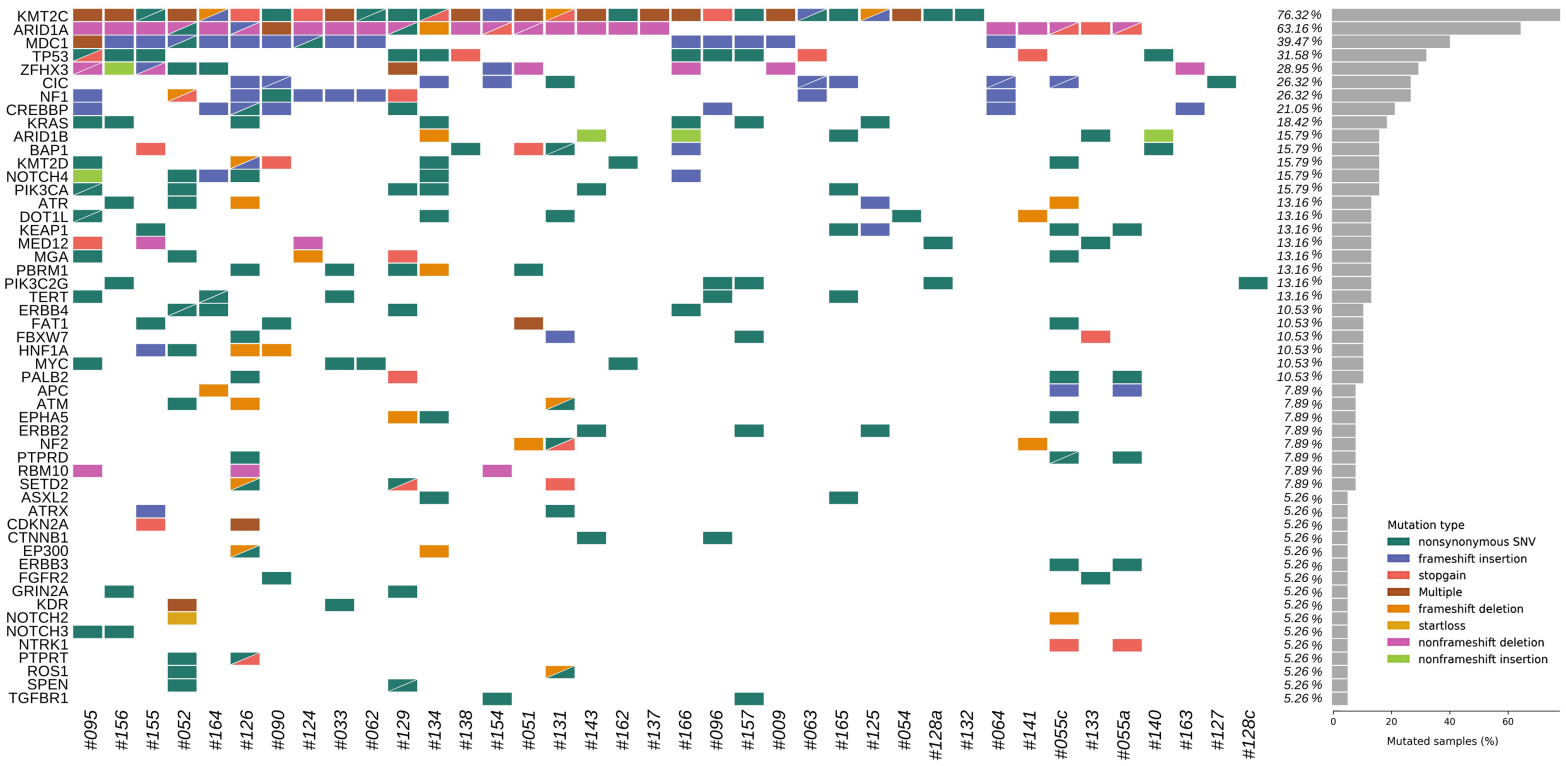
Co-mutation analysis of CUP somatic variants. CoMut plot summarizing co-occurring somatic mutations across CUP patients. Columns represent individual CUP samples, and rows correspond to mutated genes. Colored boxes indicate the presence and type of mutation. The bar plot on the right shows the percentage of samples harboring mutations in each gene.

**Figure 5:**
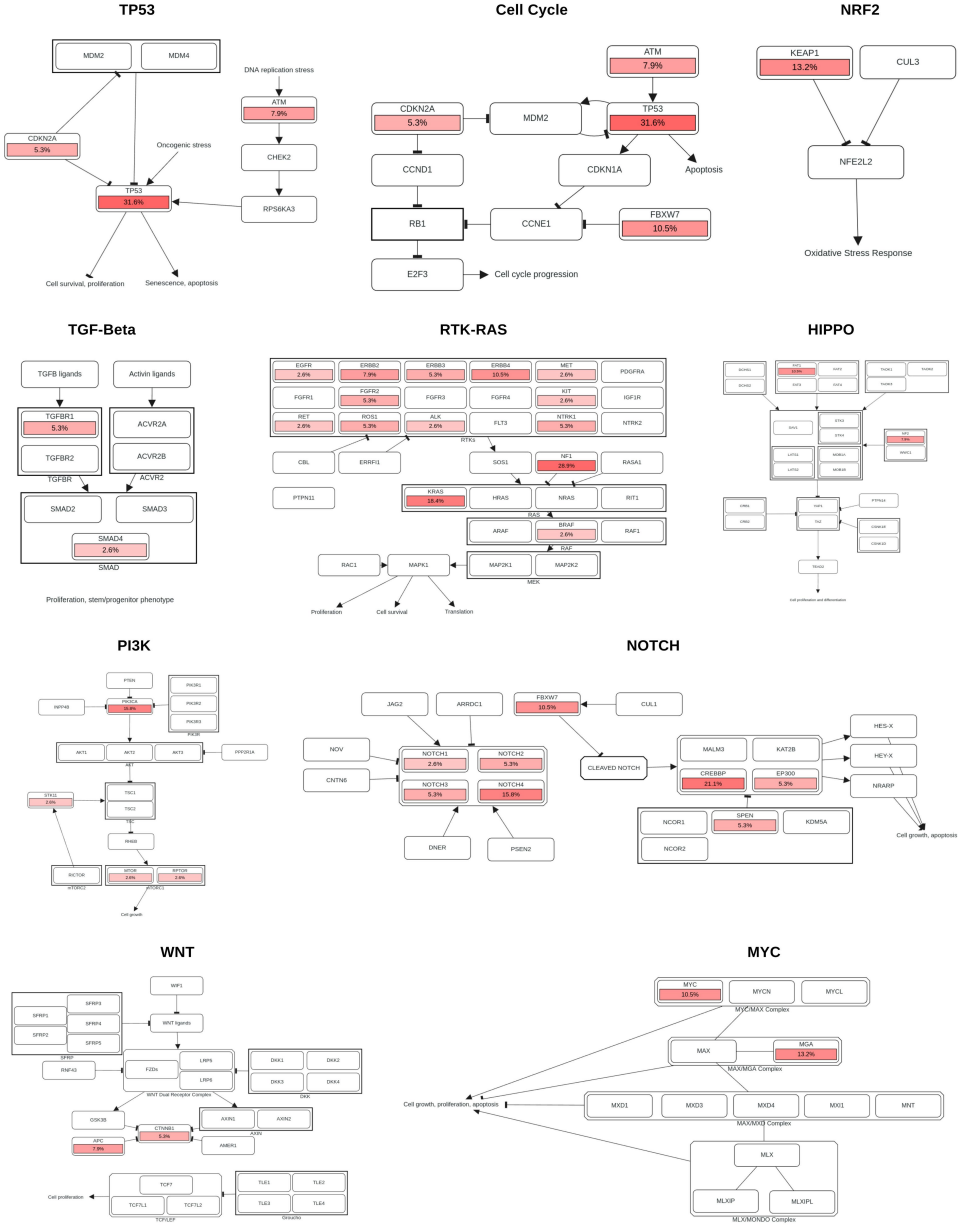
Oncogenic pathway classification of somatic mutations. Schematic representation of oncogenic pathways affected by somatic mutations in CUP patients. Genes are grouped according to their involvement in major signaling pathways. Boxes indicate the percentage of samples harboring at least one mutation in the corresponding gene. Color intensity reflects mutation frequency, with darker shades indicating higher prevalence.

Analysis of co-occurring mutations using CoMutPlot (Figure 4) revealed frequent co-alteration of KMT2C and ARID1A. Statistically significant co-occurring and mutually exclusive mutation patterns are shown in Supplementary Figure 1. Somatic mutations were classified into cancer-related pathways to characterize pathway-level alterations in CUP. Alterations in genes involved in cell-cycle regulation, NOTCH signaling, and RTK-RAS pathways were the most prevalent in this cohort (Figure 5). The prominent involvement of the NOTCH pathway may reflect the undifferentiated, stem-like phenotype of CUP, whereas alterations in RTK-RAS pathway components represent potential targets for precision therapies (Table 2).

#### Longitudinal ctDNA analysis in two CUP patients

We tracked longitudinal mutational changes at diagnosis and disease progression in two CUP patients, CUP#55 and CUP#128 (Supplementary Table 1). Both patients underwent ccfDNA sequencing to identify potentially actionable genetic alterations. Patient CUP#55, diagnosed with adenocarcinoma histotype, initially harbored an oncogenic mutation in *ARID1A*. Comprehensive immunohistochemical analysis of tumor tissue showed positivity for CK20 and CDX2. Based on these findings, together with the metastatic pattern, a gastrointestinal (GI) primary origin was suspected, and the patient was treated with tissue-based chemotherapy using a FOLFOX regimen. Upon disease progression (CUP#55c), repeated mutational analysis revealed novel likely oncogenic mutations in *ATR*, *CIC* and *NOTCH2*, along with an increased variant allele frequency of a loss-of-function mutation in *APC*.

In contrast, patient CUP#128, with squamous carcinoma histotype, did not exhibit actionable variants at baseline, although PD-L1 expression was positive. Immunohistochemical analysis demonstrated positivity for CKWS, p40, GATA-3, and p16. Based on these findings, the patient received empirical chemotherapy with carboplatin plus paclitaxel for 4 cycles, achieving a partial response. At disease progression (CUP#128c), mutational analysis showed a reduction in *PIK3C2G* mutation frequency, without the emergence of new variants. Given the positive PD-L1 expression and the interval since the last carboplatin-based treatment, second-line therapy with carboplatin plus pembrolizumab was initiated.

While the emergence of additional genetic alterations in CUP#55 suggested ongoing tumor evolution, CUP#128 displayed a more stable genomic profile with a reduction in overall mutation burden. These observations, although limited to two patients, highlight the dynamic nature of CUP tumor genomics, in which late-stage molecular changes may contribute to treatment resistance and disease progression. Ultimately, both patients developed progressive disease, and clinical deterioration limited the feasibility of further therapeutic interventions.

#### Germline variants and clonal hematopoiesis

We investigated the presence of germline genetic alterations in 39 CUP patients within the genomic regions covered by our targeted panel. After applying stringent filtering criteria, as described in the Methods section, all patients carried at least one germline variant in cancer-related genes included in the panel. These variants were reported irrespective of their predicted pathogenicity, while their clinical relevance was subsequently assessed through functional annotation. A complete list of germline variants is provided in Supplementary Table 3.

The most frequently altered genes included *KMT2C, ZFHX3, NOTCH2, SPEN, FAT1, CIC, KMT2D, MDC1, ROS1, and ARID1B*. The majority of variants were classified as benign or likely benign according to ClinGen, ClinVar, and AlphaMissense annotations, whereas a subset was categorized as pathogenic or likely pathogenic by at least two independent sources (Table 3).

**Table 3:** Pathogenic germline variants identified in CUP patients.

Samples	Gene	cDNA change	HGVSp_Short	Clinical_Significance(ClinVar)	Class (AlphaMissense)	Score (AlphaMissense)	ACMG/AMP_Pathogenic (PP3) (AlphaMissense)	Disease (ClinGen)	PS1_ID (ClinVar_ACMG)	PM5_ID (ClinVar_ACMG)
CUP#009	BAP1	c.277A>C	p.T93P		likely_pathogenic	0.9953	Strong	BAP1-related tumor predisposition syndrome		
CUP#055	NTRK1	c.2339G>C	p.R780P	Pathogenic	likely_pathogenic	0.6936		hereditary sensory and autonomic neuropathy type 4	12,303	
CUP#137	MITF	c.1255G>A	p.E318K	Pathogenic/Likely pathogenic|risk factor	likely_pathogenic	0.6129		Waardenburg syndrome type 2		995,924

Notably, the germline *MITF* p.E318K variant identified in patient CUP#137 was classified as pathogenic by both ClinVar and AlphaMissense and has been previously reported as a cancer-predisposing variant (40).

Patient CUP#55, who was diagnosed at a relatively young age (50 years), harbored a germline *NTRK1* p.R780P variant, classified as pathogenic by ClinVar and likely pathogenic by AlphaMissense. This variant met ACMG criterion PS1, as it results in the same amino acid change as a previously reported pathogenic variant (ClinVar ID: 12303). In addition, a truncating somatic mutation in the same gene (p.Q742*) (Supplementary Table 1) was detected at a variant allele frequency of 20%, suggesting potential biallelic involvement. The *NTRK1* p.R780P variant was also detected in tumor DNA at a frequency consistent with a germline origin (46%), whereas the p.Q742* mutation was confirmed in tumor tissue at a lower frequency (8%), consistent with an acquired somatic event.

One patient (CUP#009) harbored a germline *BAP1* p.T93P variant, which was classified as pathogenic according to ACMG/AMP criteria, with supporting computational evidence (PP3) based on AlphaMissense predictions.

**Figure 6:**
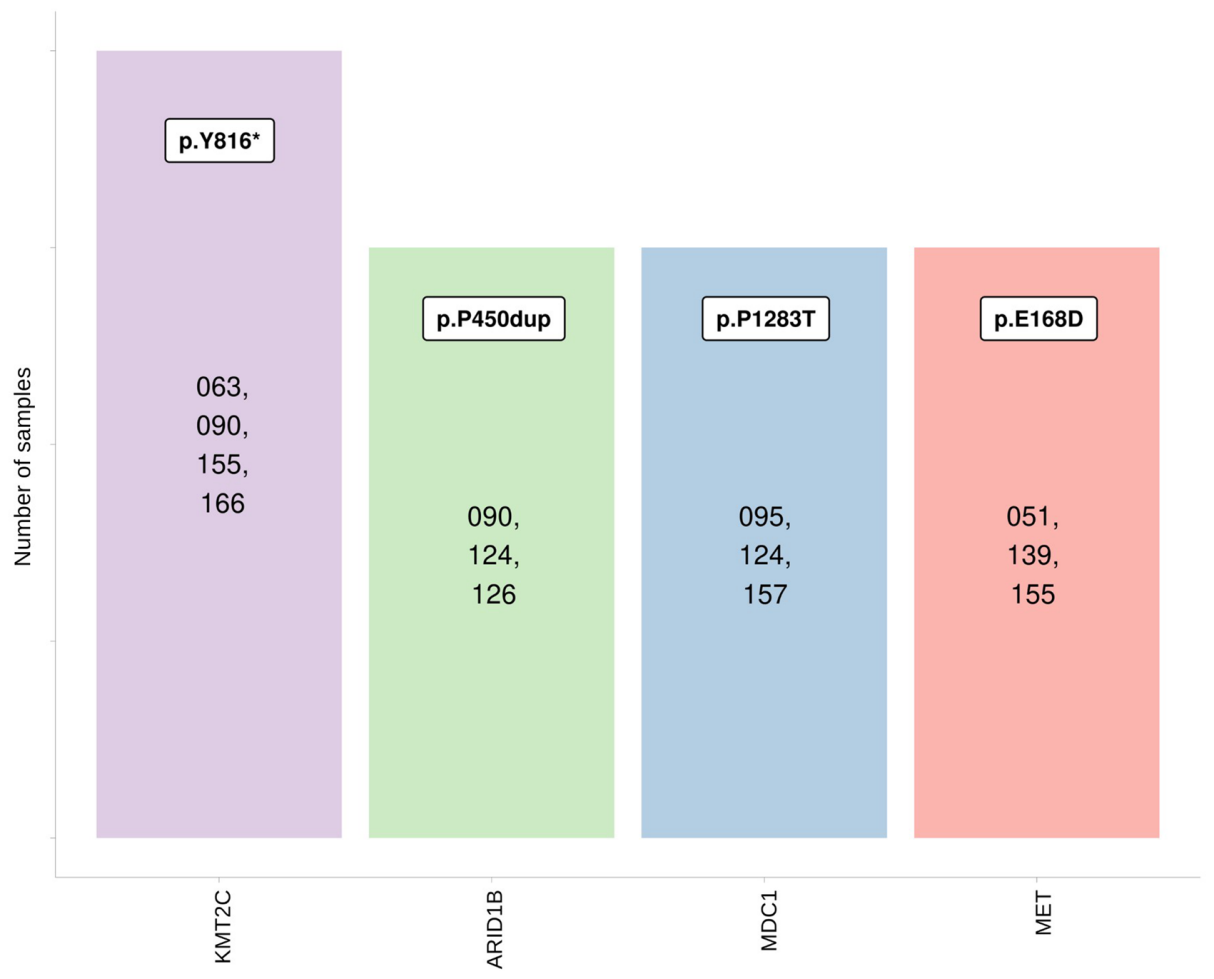
Recurrent germline variants in CUP patients. Bar plot showing non-synonymous germline variants shared by at least three unrelated CUP patients. Genes are displayed on the x-axis. Each box represents a specific germline variant, and the CUP samples carrying that variant are listed within the box. Bar height (y-axis) is proportional to the number of samples.

Among all germline variants, several non-synonymous alterations were shared by three or more unrelated CUP patients (Figure 6), suggesting the presence of recurrent germline genetic features that may warrant further investigation.

Variants affecting CHIP-associated genes (*BCOR, NF1, NOTCH2, and KMT2D*) (38, 39) were detected in PBMC-derived genomic DNA from 17 patients, with VAFs ranging from 2 to 35%, and were classified as CHIP-related (Supplementary Table 4).

#### Association with clinical outcomes

We performed an exploratory analysis to assess potential associations between clinical prognostic factors and patients’ overall survival (OS) and progression-free survival (PFS), given the limited sample size (Supplementary Figures 2, 3).

Eastern Cooperative Oncology Group (ECOG) performance status emerged as the most significant prognostic factor for both OS and PFS (*p* < 0.0001). A borderline association was observed for the neutrophil-to-lymphocyte ratio (NLR) with respect to OS (*p* = 0.075), but not PFS (*p* = 0.19). In contrast, other clinical parameters, including risk profile, histological subtype, sex, and number of metastatic sites, were not significantly associated with prognosis in our cohort.

**Figure 7:**
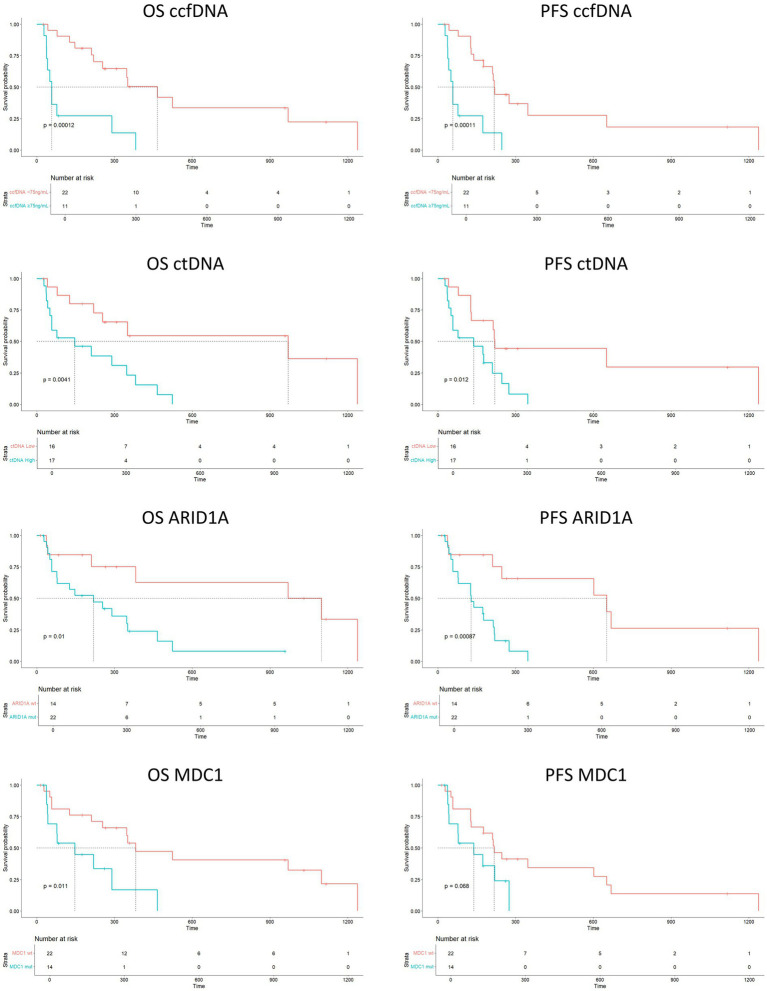
Association between molecular variables and clinical outcomes. Kaplan–Meier survival curves illustrating the association between molecular variables and clinical outcomes, including overall survival (OS) and progression-free survival (PFS). Analyses include plasma ccfDNA concentration (<75 ng/μL vs. ≥ 75 ng/μL), ctDNA fraction (low <0.1260 vs. high ≥0.1260), and mutation status (mutated vs. wild-type) of *ARID1A* and *MDC1*. *p* values were calculated using the log-rank test and are reported in each panel.

We next evaluated the univariate association between molecular variables derived from ctDNA analysis and clinical outcomes (Figure 7). Elevated plasma ccfDNA concentrations were significantly associated with poorer OS and PFS (*p* = 0.00012 and *p* = 0.00011, respectively). Similarly, a higher ctDNA fraction was associated with worse survival outcomes (OS *p* = 0.0041; PFS *p* = 0.012).

Furthermore, the presence of ctDNA mutations in ARID1A and MDC1 was associated with an unfavorable prognosis for both OS and PFS. To address the potential technical bias associated with the recurrent ARID1A Q1334del variant, survival analyses were repeated after excluding this alteration; the association with both OS and PFS remained significant (Supplementary Figure 4).

#### Identification of clinically actionable variants

We explored whether ctDNA genetic variants could represent potential targets for FDA-approved therapies, considering indications across all solid tumors due to the limited availability of data for rare cancers.

Overall, 33 patients harbored oncogenic mutations, of which 44 variants were classified as clinically relevant according to the AMP/ASCO/CAP tier system (37), as implemented in the OncoKB database (Table 2). These alterations affected genes involved in the RTK-RAS, PI3K, cell-cycle, and NOTCH pathways.

Specifically, targetable alterations were most frequently identified in *NF1* (*n* = 10 patients), *ARID1A* (*n* = 7), *KRAS* (*n* = 7), *PIK3CA* (*n* = 4), *ATR* (*n* = 3), and *FBXW7* (*n* = 3). Less frequent or patient-specific actionable alterations were observed in *ATM, CDKN2A, ERBB2, BRAF, KIT, PALB2, and STK11*.

For selected mutations in these genes, we report the therapies or therapeutic combinations supported by the highest level of evidence, as defined by OncoKB (Table 2).

## Discussion

This study provides a targeted genomic characterization of cancers of unknown primary through the integrated analysis of circulating tumor DNA (ctDNA) and matched germline DNA (gDNA) in a cohort of 39 patients, using a 92-gene panel enriched for genes frequently altered in CUP, as identified by previous comprehensive cancer panel and whole-exome sequencing studies. Our results demonstrate the feasibility and potential clinical relevance of liquid biopsy for molecular profiling of CUP, enabling the detection of both somatic and germline genetic alterations within the limits of the 92-gene panel.

Importantly, our study extends previous work by integrating ccfDNA and matched germline DNA analysis, enabling the distinction between somatic, germline, and CHIP-related variants, and providing clinically annotated variant-level data in a real-world CUP cohort. The inclusion of matched germline DNA proved to be essential for accurate variant interpretation. Although our cohort displayed marked clinical and genetic heterogeneity, we identified recurrent genetic alterations shared among multiple patients, suggesting the presence of common molecular features that may act on malignant cells of different origin, contributing to the CUP phenotype.

All patients in our cohort had sufficient circulating cell-free DNA (ccfDNA) for molecular analysis, with ctDNA accounting for up to 70% of total ccfDNA in some cases. Non-synonymous exonic variants were classified according to their origin, distinguishing somatic, germline, and clonal hematopoiesis–related mutations. The most frequently mutated somatic genes included *KMT2C, ARID1A, MDC1, TP53, HNF1A, NF1, ZFHX3, CIC, CREBBP, KRAS, ARID1B, ATR, KMT2D, NOTCH4,* and *PIK3CA*, highlighting the complex genomic landscape of CUP.

The high frequency of *KMT2C* alterations observed in our cohort is consistent with findings from large-scale cancer genomic studies and may partly reflect the gene’s large coding region. Beyond this technical consideration, *KMT2C* plays a key role in maintaining genomic stability, and its alteration has been associated with increased tumor mutational burden across several cancer types. In our study, CUP cases with higher mutational burden more frequently harbored *KMT2C* mutations, supporting a potential functional contribution of this gene to genomic instability in CUP. However, given the large coding size of KMT2C and its frequent mutation across cancer types, it is also possible that some of these alterations represent passenger events associated with increased genomic instability rather than direct drivers of tumorigenesis.

Alterations in ARID1A were also highly prevalent, with two main variants identified (R1276* and Q1334del). The elevated frequency of ARID1A alterations observed in our cohort should be interpreted with caution, as it was largely driven by the recurrent Q1334del variant located within a repetitive genomic region. Variants in such regions may be more prone to technical and analytical biases; however, the triplet deletion was validated in a subset of samples using an independent assay (data not shown). Moreover, the recurrence of this alteration across independent CUP datasets suggests potential biological relevance.

Even after excluding Q1334del, ARID1A remained among the most frequently mutated genes in our cohort, consistent with previous reports. Loss-of-function mutations in ARID1A disrupt chromatin remodeling and genomic stability and have been shown to sensitize tumor cells to BET and EZH2 inhibitors through synthetic lethality mechanisms, underscoring the potential therapeutic relevance of ARID1A alterations in CUP. Alterations in ARID1A and MDC1 may contribute to genomic instability and impaired DNA damage response, potentially explaining their association with adverse clinical outcomes, although this hypothesis requires further validation.

Frameshift mutations affecting tumor suppressor genes such as *MDC1, CIC, CREBBP,* and *NF1* were also recurrent in our cohort and are predicted to result in functional inactivation. In particular, the *NF1* frameshift mutation detected near the N-terminus is expected to cause complete loss of neurofibromin function. Previous studies have shown that patients with *NF1*-mutant solid tumors may benefit from MEK inhibitor therapy, supporting the clinical relevance of these alterations.

The *TERT* p.R672G mutation, detected in 13% of patients, is not reported in the general population, although its functional consequences remain unknown. The recurrence of this variant across multiple patients suggests a potential role in CUP pathogenesis and highlights the need for further functional validation.

A notable finding of this study is the recurrence of identical somatic mutations across unrelated patients, including alterations in *ARID1A, MDC1, CIC, CREBBP, NF1, KRAS, TERT,* and *MYC*. The presence of shared mutations supports the hypothesis that CUP may encompass recurrent molecular patterns despite clinical heterogeneity, potentially reflecting common mechanisms of tumor initiation or progression.

The clinical relevance of molecularly guided therapy in CUP is an area of active investigation, particularly for patients with unfavorable prognosis (11). In our cohort, a substantial proportion of patients harbored pathogenic or likely pathogenic alterations in genes considered actionable based on FDA-approved therapies across solid tumors, including *ARID1A, ATM, ATR, BRAF, CDKN2A, ERBB2, FGFR2, KIT, KRAS, NF1, PALB2,* and *PIK3CA*. *KRAS* mutations were detected in 18% of patients, most commonly affecting codon 12. These activating mutations are established drivers of oncogenesis and are now targetable with recently approved KRAS inhibitors, including mutation-specific agents for *KRAS* G12C. Similarly, activating *FGFR2* alterations were identified in a subset of patients, often in combination with gene amplification, supporting the potential use of FGFR inhibitors. *PIK3CA* mutations, particularly the E545K variant, were also observed and represent established therapeutic targets in several cancer types. Alterations in *ERBB2* further expand the spectrum of actionable events detected in this cohort.

Comparison with real-world diagnostic data reported in Table 2 showed that four patients (CUP#33, CUP#124, CUP#143 and CUP#051) with inadequate tumor tissue DNA for molecular testing could have had a druggable target identified through ccfDNA analysis. In addition, 14 patients harbored actionable genetic alterations that were absent from, or not detected by, the diagnostic NGS panel. Conversely, all actionable mutations (*N* = 12) identified in tumor tissue were also detected in ccfDNA, with the exception of the CTNNB1 p.D32V mutation in CUP#125.

These findings further support the relevance of liquid biopsy–based precision oncology approaches in CUP, particularly in settings where standard therapeutic options are limited, in agreement with previous studies (21, 41).

Pathway-level analysis revealed frequent involvement of RTK-RAS and NOTCH signaling pathways. The prominence of RTK-RAS alterations aligns with evidence from the CUPISCO trial, which demonstrated clinical benefit from RTK-targeted therapies in CUP patients (11). The extent of NOTCH pathway involvement was an unexpected finding. Although NOTCH signaling can exert context-dependent effects in cancer, its frequent alteration in CUP may reflect the undifferentiated and aggressive phenotype characteristic of these tumors. NOTCH signaling plays a central role in cell fate determination, cancer stem cell maintenance, angiogenesis, and metastasis, and its dysregulation may contribute to early dissemination and therapy resistance in CUP.

Longitudinal ctDNA analysis in two patients provided insights into tumor evolution during disease progression. In one case, the acquisition of additional pathogenic alterations suggested ongoing clonal evolution and potential mechanisms of treatment resistance, whereas in the other case, a reduction in mutation burden and ctDNA levels was observed, likely reflecting therapeutic response. These findings highlight the dynamic nature of CUP genomics and support the use of liquid biopsy for disease monitoring.

An important aspect of this study is the evaluation of clonal hematopoiesis of indeterminate potential (CHIP) (38). We identified CHIP-related mutations in approximately half of the cohort, a prevalence higher than that typically reported in solid tumors. Although our panel did not include canonical CHIP driver genes, mutations in CHIP-associated genes *BCOR, NF1, NOTCH2,* and *KMT2D* were detected in PBMC-derived gDNA at VAFs consistent with clonal hematopoiesis. These findings underscore the importance of sequencing matched blood-derived DNA to avoid misclassification of CHIP-related variants as tumor-derived alterations.

Finally, the analysis of germline variants identified pathogenic alterations in three patients, involving *NTRK1, MITF, and BAP1*. Germline MITF variants, particularly p.E318K, are established moderate-risk alleles for melanoma and have been associated with renal cell carcinoma, whereas evidence for other tumor types, including sarcomas, remains limited to sporadic reports without a confirmed causal role (40, 42–44). The patient harboring this variant developed a CUP with leiomyosarcoma histology, and no additional somatic oncogenic alterations were detected using our panel.

The coexistence of a germline NTRK1 mutation and a somatic second hit in the same gene in one patient suggests a possible hereditary contribution in a subset of CUP cases.

In addition, one patient harbored a germline BAP1 variant classified as pathogenic according to ACMG/AMP criteria. Given the established role of BAP1 in tumor predisposition syndromes, this finding may warrant further investigation, although its contribution to CUP remains to be defined.

Furthermore, several germline variants of uncertain significance were shared among multiple unrelated patients, raising the possibility of underlying genetic susceptibility or modifying factors that warrant further investigation in larger cohorts.

In conclusion, our findings demonstrate that liquid biopsy–based targeted sequencing provides valuable insights into the genomic landscape of CUP, revealing recurrent somatic alterations, clinically actionable mutations, germline variants, and a high prevalence of clonal hematopoiesis. The integration of ctDNA and matched gDNA analysis is critical for accurate variant interpretation and supports the application of precision oncology strategies in CUP.

## Data Availability

Sequencing data have been deposited in the European Nucleotide Archive (ENA) at EMBL-EBI under accession number PRJEB85169 (currently under private status). Due to privacy and sensitivity considerations, raw sequencing data supporting the findings of this study are not publicly available but may be obtained from the corresponding author upon reasonable request. Processed variant-level sequencing data are provided in the Supplementary material.

## Footnotes

1
https://github.com/oncokb/oncokb-annotator

## Supplementary material

SUPPLEMENTARY FIGURE 1 Co-occurrence and mutual exclusivity of somatic mutations. Graph illustrating patterns of co-occurring and mutually exclusive somatic mutations. Statistically significant associations are indicated (***p* < 0.05, **p* < 0.1).

SUPPLEMENTARY FIGURE 2 Association of clinico-pathological variables with overall survival. Kaplan–Meier survival curves showing the univariate association between clinico-pathological variables and overall survival (OS). Variables analyzed include ESMO cancer risk profile (favorable vs. unfavorable), histology (adenocarcinoma vs. others), sex (female vs. male), number of metastatic sites (1, 2, or ≥3), neutrophil-to-lymphocyte ratio (NLR <5 vs. ≥5), and ECOG performance status (0, 1, 2, or 3). Log-rank *p* values are reported.

SUPPLEMENTARY FIGURE 3 Association of clinico-pathological variables with progression-free survival. Kaplan–Meier survival curves illustrating the univariate association between clinico-pathological variables and progression-free survival (PFS), including ESMO cancer risk profile (favorable vs. unfavorable), histology (adenocarcinoma vs. others), sex (female vs. male), number of metastatic sites (1, 2, or ≥3), neutrophil-to-lymphocyte ratio (NLR <5 vs. ≥5), and ECOG performance status (0, 1, 2, or 3). Log-rank *P* values are reported.

SUPPLEMENTARY FIGURE 4 Kaplan–Meier survival analysis excluding the ARID1A Q1334del variant. Kaplan–Meier curves showing overall survival (OS) and progression-free survival (PFS) according to ARID1A mutation status after excluding the recurrent Q1334del variant. Patients were stratified based on the presence or absence of ARID1A mutations other than Q1334del. Survival differences between groups were assessed using the log-rank test, and corresponding p-values are reported.

## References

[1] Kramer A Bochtler T Pauli C Baciarello G Delorme S Hemminki K . Cancer of unknown primary: ESMO clinical practice guideline for diagnosis, treatment and follow-up. Ann Oncol. (2023) 34:228–46. doi: 10.1016/j.annonc.2022.11.013, 36563965

[2] Laprovitera N Riefolo M Ambrosini E Klec C Pichler M Ferracin M. Cancer of unknown primary: challenges and progress in clinical management. Cancers. (2021) 13:451. doi: 10.3390/cancers13030451, 33504059 PMC7866161

[3] Pavlidis N Briasoulis E Hainsworth J Greco FA. Diagnostic and therapeutic management of cancer of an unknown primary. Eur J Cancer. (2003) 39:1990–2005. doi: 10.1016/S0959-8049(03)00547-1, 12957453

[4] Hermans K Kazemzadeh F Loef C Jansen RLH Nagtegaal ID van den Brandt PA . Risk factors for cancer of unknown primary: a literature review. BMC Cancer. (2023) 23:314. doi: 10.1186/s12885-023-10794-6 37020279 PMC10077635

[5] Ettinger DS Handorf CR Agulnik M Bowles DW Cates JM Cristea M . Occult primary, version 3.2014. J Natl Compr Cancer Netw. (2014) 12:969–74. doi: 10.6004/jnccn.2014.0093, 24994917

[6] NCCN. (n.d.) Occult Primary (Version 1.2026). Available online at: https://www.nccn.org/professionals/physician_gls/pdf/occult_blocks.pdf (Accessed March 27, 2026).

[7] Raghav K Hwang H Jacome AA Bhang E Willett A Huey RW . Development and validation of a novel nomogram for individualized prediction of survival in Cancer of unknown primary. Clin Cancer Res. (2021) 27:3414–21. doi: 10.1158/1078-0432.CCR-20-4117, 33858857 PMC8197749

[8] Pavlidis N Pentheroudakis G. Cancer of unknown primary site. Lancet. (2012) 379:1428–35. doi: 10.1016/S0140-6736(11)61178-1, 22414598

[9] Pouyiourou M Kraft BN Wohlfromm T Stahl M Kubuschok B Loffler H . Nivolumab and ipilimumab in recurrent or refractory cancer of unknown primary: a phase II trial. Nat Commun. (2023) 14:6761. doi: 10.1038/s41467-023-42400-5, 37875494 PMC10598029

[10] Yulian ED Hwei LRY Tambun R Siswoyo AD Ham MF Suroyo I. Comprehensive evaluation on cancer of unknown primary site and how we managed it: a case report. Int J Surg Case Rep. (2022) 93:106954. doi: 10.1016/j.ijscr.2022.106954, 35339815 PMC8961183

[11] Kramer A Bochtler T Pauli C Shiu KK Cook N de Menezes JJ . Molecularly guided therapy versus chemotherapy after disease control in unfavourable cancer of unknown primary (CUPISCO): an open-label, randomised, phase 2 study. Lancet. (2024) 404:527–39. doi: 10.1016/S0140-6736(24)00814-6, 39096924

[12] Fuentes Bayne HE Kasi PM Ma L Hart LL Wong J Spigel DR . Personalized therapy selection by integration of molecular Cancer classification by the 92-gene assay and tumor profiling in patients with Cancer of unknown primary. JCO Precis Oncol. (2024) 8:e2400191. doi: 10.1200/PO.24.00191, 39231374 PMC11382827

[13] Hainsworth JD Rubin MS Spigel DR Boccia RV Raby S Quinn R . Molecular gene expression profiling to predict the tissue of origin and direct site-specific therapy in patients with carcinoma of unknown primary site: a prospective trial of the Sarah Cannon research institute. J Clin Oncol. (2013) 31:217–23. doi: 10.1200/JCO.2012.43.3755, 23032625

[14] Varadhachary GR Spector Y Abbruzzese JL Rosenwald S Wang H Aharonov R . Prospective gene signature study using microRNA to identify the tissue of origin in patients with carcinoma of unknown primary. Clin Cancer Res. (2011) 17:4063–70. doi: 10.1158/1078-0432.CCR-10-2599, 21531815

[15] Laprovitera N Riefolo M Porcellini E Durante G Garajova I Vasuri F . MicroRNA expression profiling with a droplet digital PCR assay enables molecular diagnosis and prognosis of cancers of unknown primary. Mol Oncol. (2021) 15:2732–51. doi: 10.1002/1878-0261.13026, 34075699 PMC8486570

[16] Moran S Martinez-Cardus A Sayols S Musulen E Balana C Estival-Gonzalez A . Epigenetic profiling to classify cancer of unknown primary: a multicentre, retrospective analysis. Lancet Oncol. (2016) 17:1386–95. doi: 10.1016/S1470-2045(16)30297-2, 27575023

[17] Conway AM Pearce SP Clipson A Hill SM Chemi F Slane-Tan D . A cfDNA methylation-based tissue-of-origin classifier for cancers of unknown primary. Nat Commun. (2024) 15:3292. doi: 10.1038/s41467-024-47195-7, 38632274 PMC11024142

[18] Nguyen L Van Hoeck A Cuppen E. Machine learning-based tissue of origin classification for cancer of unknown primary diagnostics using genome-wide mutation features. Nat Commun. (2022) 13:4013. doi: 10.1038/s41467-022-31666-w, 35817764 PMC9273599

[19] Schipper LJ Samsom KG Snaebjornsson P Battaglia T Bosch LJW Lalezari F . Complete genomic characterization in patients with cancer of unknown primary origin in routine diagnostics. ESMO Open. (2022) 7:100611. doi: 10.1016/j.esmoop.2022.100611, 36463731 PMC9808446

[20] Rebello RJ Posner A Dong R Prall OWJ Sivakumaran T Mitchell CB . Whole genome sequencing improves tissue-of-origin diagnosis and treatment options for cancer of unknown primary. Nat Commun. (2025) 16:4422. doi: 10.1038/s41467-025-59661-x, 40393956 PMC12092688

[21] Kato S Weipert C Gumas S Okamura R Lee S Sicklick JK . Therapeutic Actionability of circulating cell-free DNA alterations in carcinoma of unknown primary. JCO Precis Oncol. (2021) 5:1687–98. doi: 10.1200/PO.21.00011, 34778692 PMC8585281

[22] Jaiswal S. Clonal hematopoiesis and nonhematologic disorders. Blood. (2020) 136:1606–14. doi: 10.1182/blood.2019000989, 32736379 PMC8209629

[23] Jaiswal S Ebert BL. Clonal hematopoiesis in human aging and disease. Science. (2019) 366:aan4673. doi: 10.1126/science.aan4673, 31672865 PMC8050831

[24] An Y Guan Y Xu Y Han Y Wu C Bao C . The diagnostic and prognostic usage of circulating tumor DNA in operable hepatocellular carcinoma. Am J Transl Res. (2019) 11:6462–74. 31737198 PMC6834494

[25] Consortium APG. AACR project GENIE: powering precision medicine through an international consortium. Cancer Discov. (2017) 7:818–31. doi: 10.1158/2159-8290.CD-17-0151, 28572459 PMC5611790

[26] Zehir A Benayed R Shah RH Syed A Middha S Kim HR . Mutational landscape of metastatic cancer revealed from prospective clinical sequencing of 10,000 patients. Nat Med. (2017) 23:703–13. doi: 10.1038/nm.4333, 28481359 PMC5461196

[27] Laprovitera N Salamon I Gelsomino F Porcellini E Riefolo M Garonzi M . Genetic characterization of Cancer of unknown primary using liquid biopsy approaches. Front Cell Dev Biol. (2021) 9:666156. doi: 10.3389/fcell.2021.666156, 34178989 PMC8222689

[28] Wang K Li M Hakonarson H. ANNOVAR: functional annotation of genetic variants from high-throughput sequencing data. Nucleic Acids Res. (2010) 38:e164. doi: 10.1093/nar/gkq603, 20601685 PMC2938201

[29] Debyani C. (n.d.) OncoKB: A Precision Oncology Knowledge Base. Available online at: https://www.oncokb.org/ (Accessed May 16, 2017)

[30] Crowdis J He MX Reardon B Van Allen EM. CoMut: visualizing integrated molecular information with comutation plots. Bioinformatics. (2020) 36:4348–9. doi: 10.1093/bioinformatics/btaa554, 32502231 PMC7520041

[31] Bahceci I Dogrusoz U La KC Babur O Gao J Schultz N. PathwayMapper: a collaborative visual web editor for cancer pathways and genomic data. Bioinformatics. (2017) 33:2238–40. doi: 10.1093/bioinformatics/btx149, 28334343 PMC5859976

[32] Chao K. (n.d.) gnomAD v4.0. Available online at: https://gnomad.broadinstitute.org/ (Accessed November 1, 2023).

[33] Schlegelberger B Mecucci C Wlodarski M. Review of guidelines for the identification and clinical care of patients with genetic predisposition for hematological malignancies. Fam Cancer. (2021) 20:295–303. doi: 10.1007/s10689-021-00263-z, 34057692 PMC8484082

[34] Brown DW Fine AD Sun D Tukachinsky H McDevitt M Pontbriand K . Abstract 1959: variant origin prediction (VOP) determines the tumor-somatic, germline, or clonal hematopoiesis (CH) origin of short variants (SVs) detected in liquid biopsy (LBx). Cancer Res. (2025) 85:1959. doi: 10.1158/1538-7445.AM2025-1959, 36230740

[35] Richards S Aziz N Bale S Bick D Das S Gastier-Foster J . Standards and guidelines for the interpretation of sequence variants: a joint consensus recommendation of the American College of Medical Genetics and Genomics and the Association for Molecular Pathology. Genet Med. (2015) 17:405–24. doi: 10.1038/gim.2015.30, 25741868 PMC4544753

[36] Horak P Griffith M Danos AM Pitel BA Madhavan S Liu X . Standards for the classification of pathogenicity of somatic variants in cancer (oncogenicity): joint recommendations of clinical genome resource (ClinGen), Cancer genomics consortium (CGC), and variant interpretation for Cancer consortium (VICC). Genet Med. (2022) 24:986–98. doi: 10.1016/j.gim.2022.01.001, 35101336 PMC9081216

[37] Li MM Datto M Duncavage EJ Kulkarni S Lindeman NI Roy S . Standards and guidelines for the interpretation and reporting of sequence variants in Cancer: a joint consensus recommendation of the Association for Molecular Pathology, American Society of Clinical Oncology, and College of American Pathologists. J Mol Diagn. (2017) 19:4–23. doi: 10.1016/j.jmoldx.2016.10.002, 27993330 PMC5707196

[38] Jaiswal S Natarajan P Silver AJ Gibson CJ Bick AG Shvartz E . Clonal hematopoiesis and risk of atherosclerotic cardiovascular disease. N Engl J Med. (2017) 377:111–21. doi: 10.1056/NEJMoa1701719, 28636844 PMC6717509

[39] Vlasschaert C Mack T Heimlich JB Niroula A Uddin MM Weinstock J . A practical approach to curate clonal hematopoiesis of indeterminate potential in human genetic data sets. Blood. (2023) 141:2214–23. doi: 10.1182/blood.2022018825, 36652671 PMC10273159

[40] Guhan SM Artomov M McCormick S Njauw C Stratigos AJ Shannon K . Cancer risks associated with the germline MITF(E318K) variant. Sci Rep. (2020) 10:17051. doi: 10.1038/s41598-020-74237-z, 33051548 PMC7555480

[41] Ross JS Wang K Gay L Otto GA White E Iwanik K . Comprehensive genomic profiling of carcinoma of unknown primary site: new routes to targeted therapies. JAMA Oncol. (2015) 1:40–9. doi: 10.1001/jamaoncol.2014.216, 26182302

[42] Brohl AS Patidar R Turner CE Wen X Song YK Wei JS . Frequent inactivating germline mutations in DNA repair genes in patients with Ewing sarcoma. Genet Med. (2017) 19:955–8. doi: 10.1038/gim.2016.206, 28125078 PMC5529247

[43] Carvalho NA Santiago KM Maia JML Costa FD Formiga MN Soares DCQ . Prevalence and clinical implications of germline pathogenic variants in cancer predisposing genes in young patients across sarcoma subtypes. J Med Genet. (2023) 61:61–8. doi: 10.1136/jmg-2023-109269, 37536918 PMC10803955

[44] Davis IJ Kim JJ Ozsolak F Widlund HR Rozenblatt-Rosen O Granter SR . Oncogenic MITF dysregulation in clear cell sarcoma: defining the MiT family of human cancers. Cancer Cell. (2006) 9:473–84. doi: 10.1016/j.ccr.2006.04.021, 16766266

