# Supplementary figures and images for: Cancer of unknown primary genomic profiling from cell-free DNA provides insights into CUP biology and vulnerabilities
Source: Front Med (Lausanne). 2026 Jun 3;13:1777582. doi: 10.3389/fmed.2026.1777582 (PMC13271982; doi:10.3389/fmed.2026.1777582)

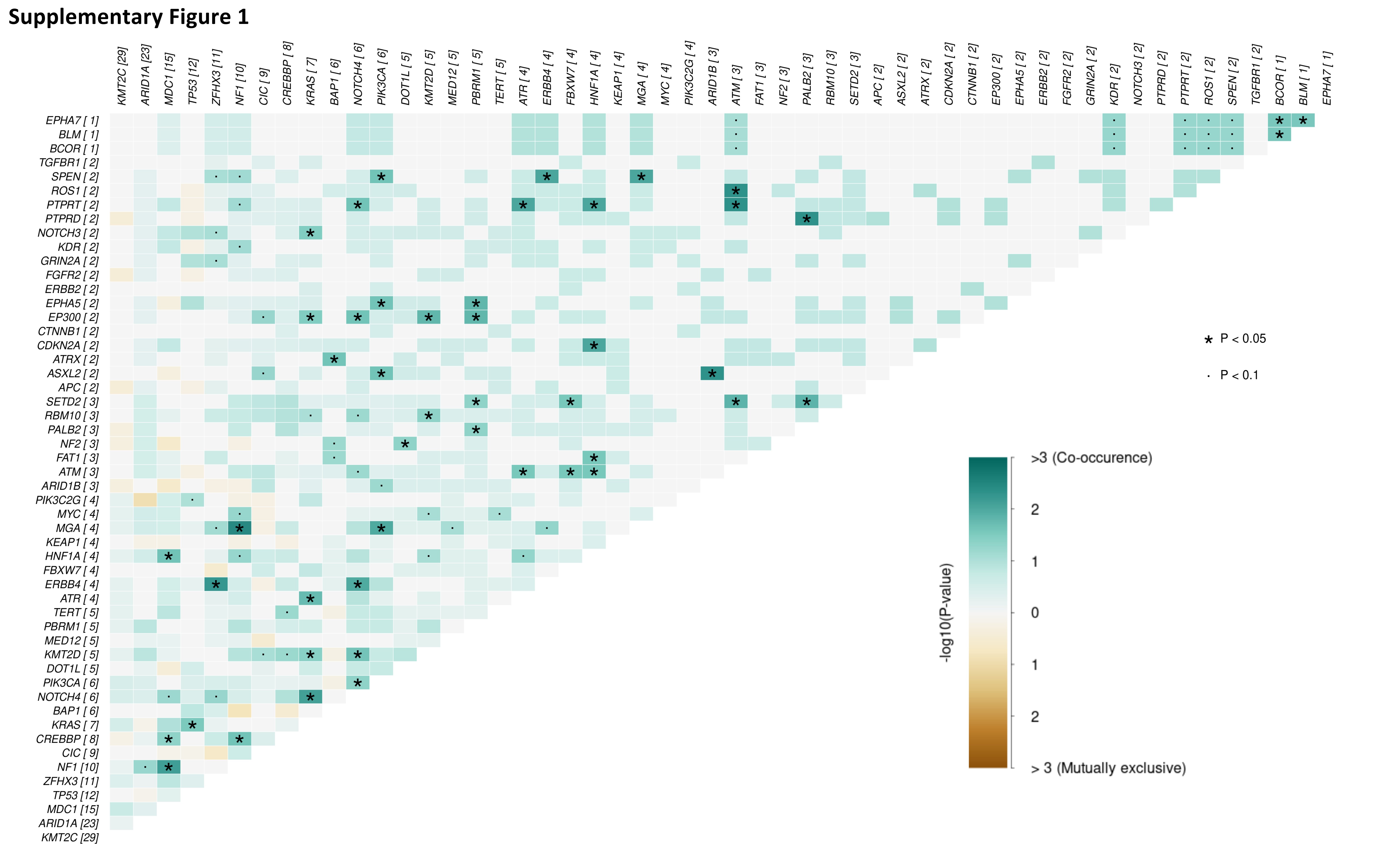

Supplement: SUPPLEMENTARY FIGURE 1 — Co-occurrence and mutual exclusivity of somatic mutations. Graph illustrating patterns of co-occurring and mutually exclusive somatic mutations. Statistically significant associations are indicated (**p < 0.05, *p < 0.1). [file Image_1.jpeg]

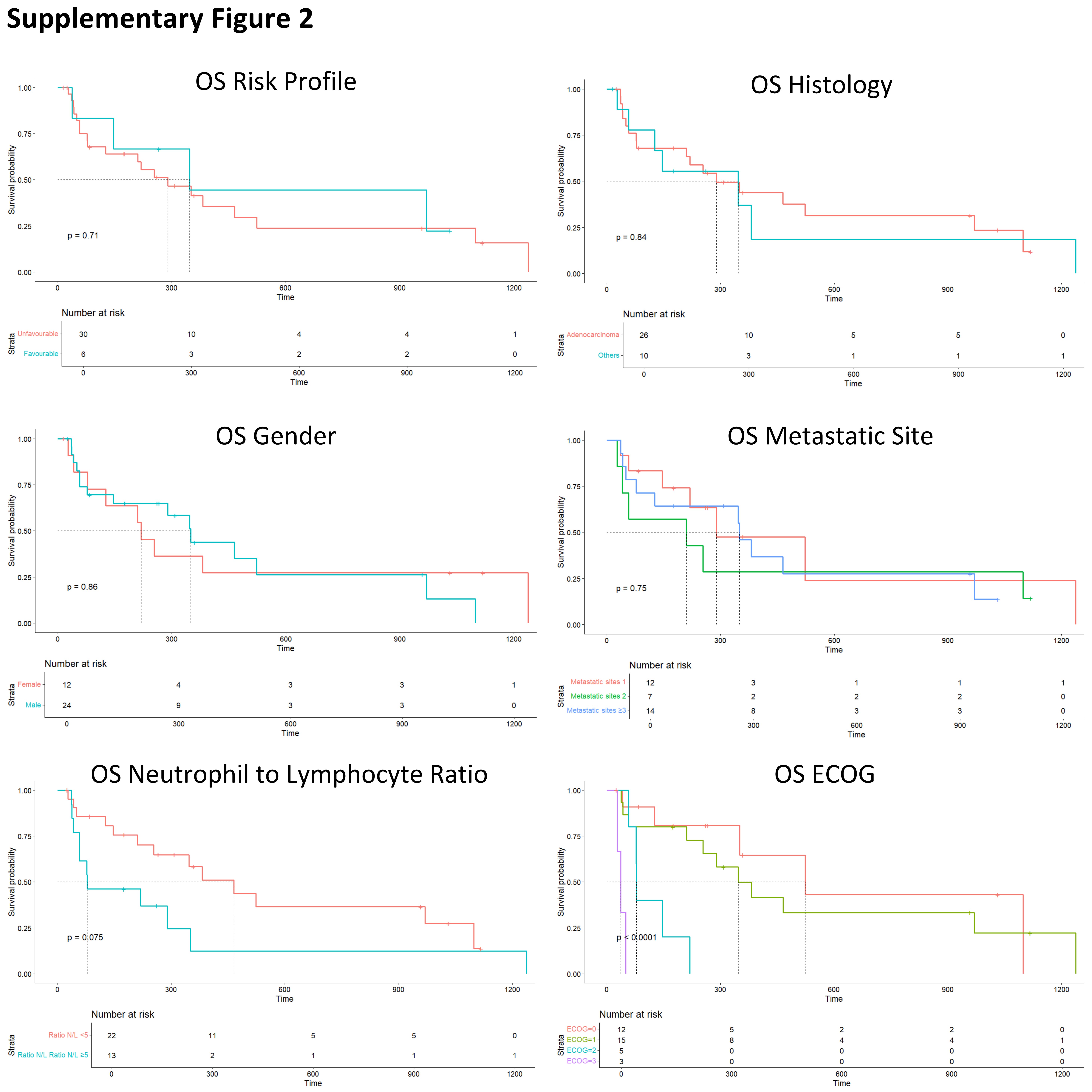

Supplement: SUPPLEMENTARY FIGURE 2 — Association of clinico-pathological variables with overall survival. Kaplan–Meier survival curves showing the univariate association between clinico-pathological variables and overall survival (OS). Variables analyzed include ESMO cancer risk profile (favorable vs. unfavorable), histology (adenocarcinoma vs. others), sex (female vs. male), number of metastatic sites (1, 2, or ≥3), neutrophil-to-lymphocyte ratio (NLR <5 vs. ≥5), and ECOG performance status (0, 1, 2, or 3). Log-rank p values are reported. [file Image_2.jpeg]

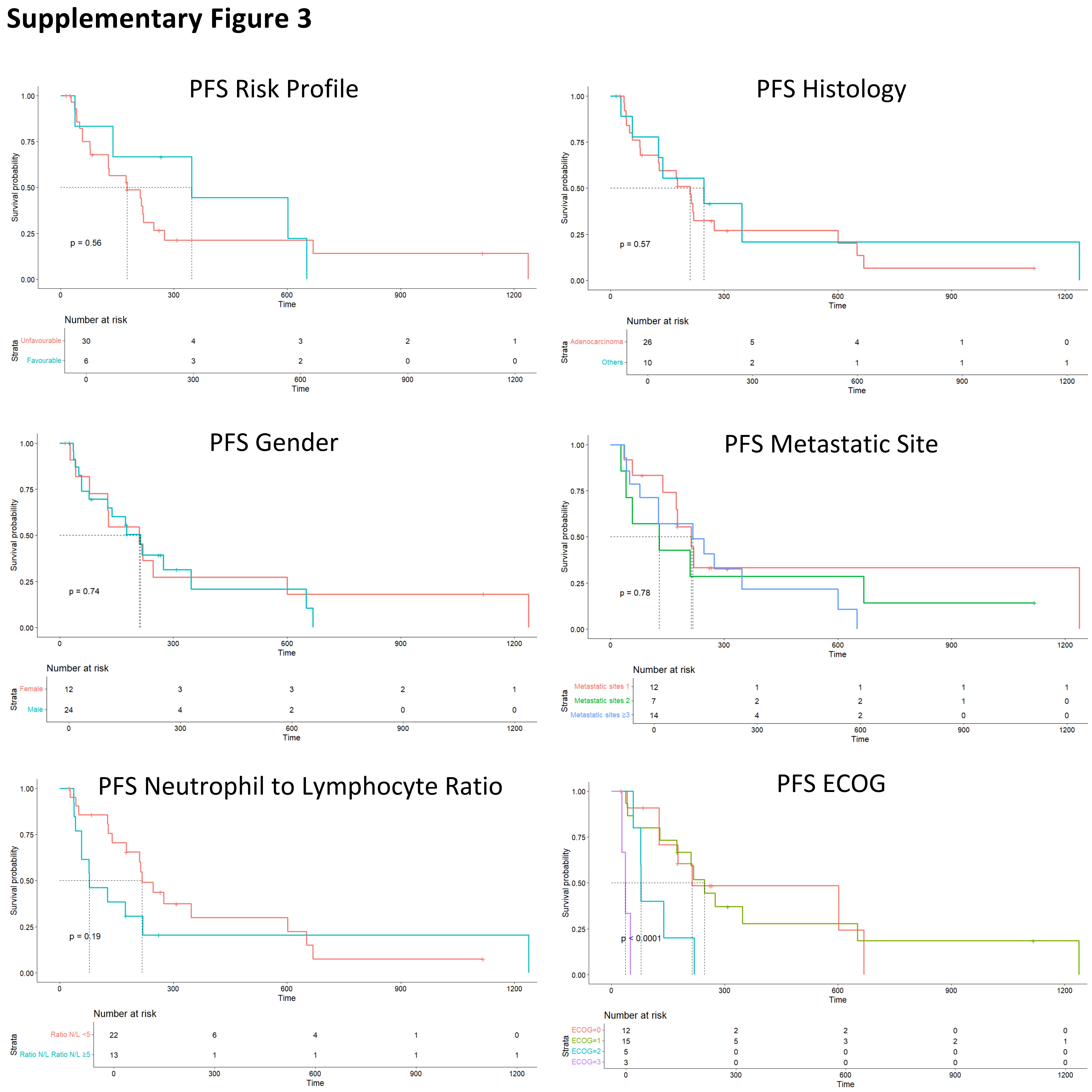

Supplement: SUPPLEMENTARY FIGURE 3 — Association of clinico-pathological variables with progression-free survival. Kaplan–Meier survival curves illustrating the univariate association between clinico-pathological variables and progression-free survival (PFS), including ESMO cancer risk profile (favorable vs. unfavorable), histology (adenocarcinoma vs. others), sex (female vs. male), number of metastatic sites (1, 2, or ≥3), neutrophil-to-lymphocyte ratio (NLR <5 vs. ≥5), and ECOG performance status (0, 1, 2, or 3). Log-rank P values are reported. [file Image_3.jpeg]

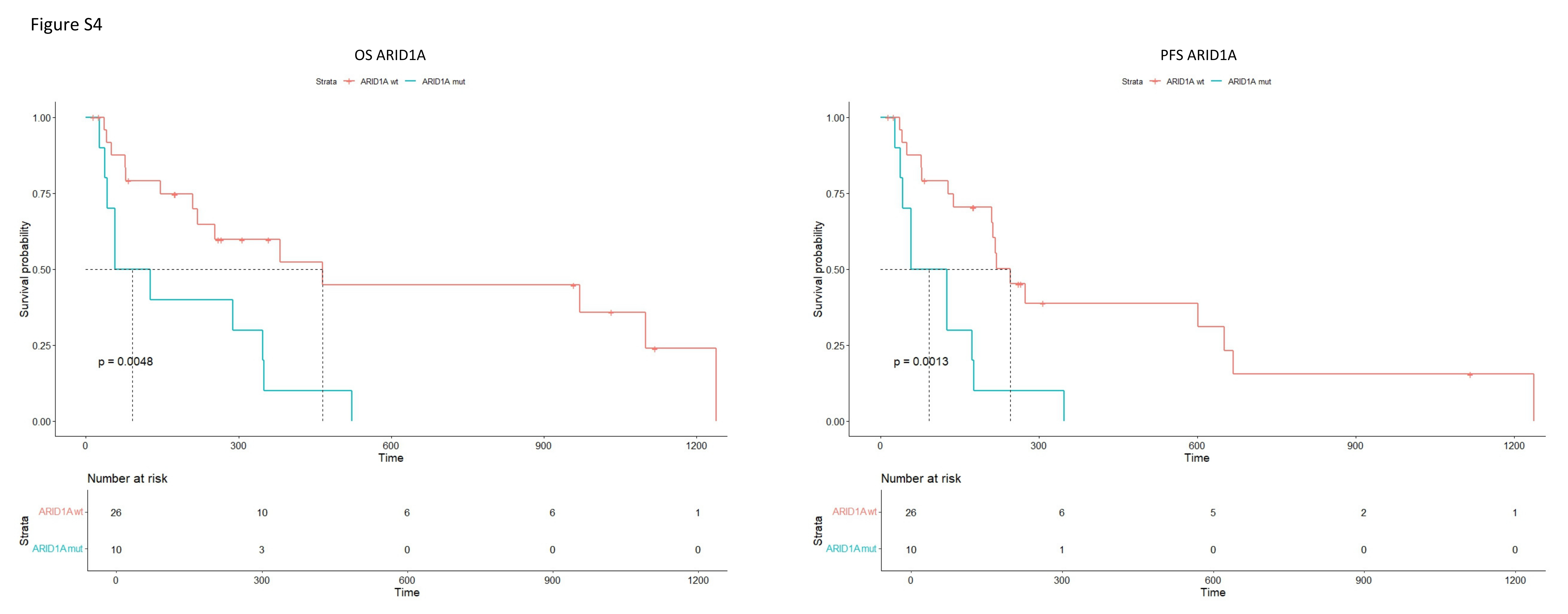

Supplement: SUPPLEMENTARY FIGURE 4 — Kaplan–Meier survival analysis excluding the ARID1A Q1334del variant. Kaplan–Meier curves showing overall survival (OS) and progression-free survival (PFS) according to ARID1A mutation status after excluding the recurrent Q1334del variant. Patients were stratified based on the presence or absence of ARID1A mutations other than Q1334del. Survival differences between groups were assessed using the log-rank test, and corresponding p-values are reported. [file Image_4.png]
